# Shuanghuanglian oral preparations combined with azithromycin for treatment of Mycoplasma pneumoniae pneumonia in Asian children: A systematic review and meta-analysis of randomized controlled trials

**DOI:** 10.1371/journal.pone.0254405

**Published:** 2021-07-13

**Authors:** Yingying Peng, Zhe Chen, Yanjiao Li, Qiu Lu, Huanmin Li, Yaowei Han, Dan Sun, Xinmin Li

**Affiliations:** 1 Department of Pediatrics, First Teaching Hospital of Tianjin University of Traditional Chinese Medicine, Tianjin, China; 2 National Clinical Research Center for Chinese Medicine Acupuncture and Moxibustion, Tianjin, China; 3 Evidence-based Medicine Center, Tianjin University of Traditional Chinese Medicine, Tianjin, China; University of Pittsburgh School of Medicine, UNITED STATES

## Abstract

**Background:**

Mycoplasma pneumoniae is one of the main causes of community-acquired pneumonia. Due to the imperfect immune system of children, this also causes Mycoplasma pneumoniae pneumonia (MPP) to be more common in children. Globally, the incidence of MPP in children is gradually increasing. This study was the first to systematically review the clinical efficacy and safety of Shuanghuanglian (SHL) oral preparations combined with azithromycin in the treatment of MPP in children.

**Methods:**

This study fully retrieved 3 Chinese databases and 5 English databases to search the randomized controlled trials (RCTs) of SHL oral preparations combined with azithromycin in the treatment of children with MPP. The search time is from the inception to September 2020. Data extraction and risk bias evaluation were performed independently by two researchers. We conducted a Meta-analysis of all the outcome indicators. Besides, Meta-regression, subgroup analysis, and heterogeneity analysis were used for the primary outcomes to find the possible potential confounding factors.

**Results:**

Finally, we included 27 RCTs involving 2884 patients. SHL oral preparations combined with azithromycin were better than azithromycin alone in response rate (RR = 1.14, 95% CI[1.11, 1.18]; low certainty evidence), disappearance time of fever(MD = -1.72, 95% CI[-2.47, -0.97]; low certainty evidence), disappearance time of cough (MD = -2.95, 95% CI[-3.55, -2.34]; low certainty evidence), and disappearance time of pulmonary rales (MD = -2.13, 95% CI[-2.88, -1.38]; low certainty evidence). The Meta-regression results showed that the course of disease, age, and method of administration may be the source of heterogeneity. Subgroup analysis and sensitivity analysis have found that the results were stable. For other related clinical symptoms, T lymphocytes, and Serum inflammatory factors, SHL oral preparations combined with azithromycin was better than azithromycin alone, and the difference was statistically significant. For adverse events with low certainty evidence, safety needs further verification.

**Conclusion:**

Based on the results of meta-analysis with low certainty evidence, we believed that SHL oral preparations combined with azithromycin likely be effectively improved clinical symptoms compared with azithromycin alone. Low certainty evidence showed that SHL may safety with no serious adverse events. Due to these limitations, the safety needs further verification. More high-quality, multicenter, and large-sample RCTs should be tested and verified in the future.

## Introduction

Mycoplasma pneumoniae (MP), whose volume is between bacteria and viruses, is the smallest prokaryote without a cell wall, and it is a common cause of upper and lower respiratory infections [1, 2]. MP is one of the most common causes of community-acquired pneumonia (CAP) in children, especially in those over 5 years of age [3]. Although the mechanisms of Mycoplasma pneumoniae pneumonia (MPP) have been constantly researched, its pathogenesis has yet to be completely elucidated. Children are more sensitive to exposure to the MP infection as their bodies are in a stage of rapid development and their immune systems are insufficiently mature [4]. MPP accounts for about 40% of CAP in children, and its incidence is on the increase and has become a significant health problem globally [5, 6]. The main clinical symptoms of MPP are dry cough, fever, nervous tension, headache, and other symptoms, and it may not be easily recognized for clinicians to make the clinical diagnosis by these subtle symptoms [7]. Based on the complexity of clinical diagnosis, MPP is often confused and misdiagnosed with viral and bacterial respiratory tract infections [1].

At the present stage, macrolide antibiotics are widely used in the clinical treatment of MPP in children [1]. Among macrolide antibiotics, azithromycin has proven to be an effective treatment and has been recommended as first-line drugs [8]. Azithromycin has significant short-term efficacy for children with MPP, but it can cause adverse effects and complications of digestive and nervous systems [9]. With the long-term use of azithromycin, azithromycin resistance has now emerged as a significant clinical health problem in children, which may lead to a large number of extrapulmonary complications and severe clinical symptoms [1, 10, 11]. Since macrolide-resistant strains were isolated from patients with MPP in Japan, the problem of macrolide-resistance has been receiving more attention in many countries [12]. The incidence of macrolide-resistance makes up approximately 80% in Asia, while the incidence in Europe and the United States remains at a low level [13–16].

Traditional Chinese medicines (TCMs) have been known for centuries and accumulation of experience in the treatment of pneumonia [17]. Based on these advantages, TCMs have become the focus of scientific research in China, and have confirmed by non-directional and multi-target to regulate inflammatory cytokine, improve the immune system, reduce apoptosis, and so on [18]. Among all treatments of TCMs, traditional Chinese patent medicines (TCPMs) are well-established preparations, which have been approved and produced by the National Medical Products Administration. Shuanghuanglian (SHL) oral preparations were the common TCPMs for children with MPP, and has the effect of broad-spectrum antivirus and improving immunologic function [19], involving that SHL granules, SHL oral liquid, and so on. In China, SHL oral preparations are often widely used in combination with azithromycin and other macrolide antibiotics and have performed a better clinical therapeutic effect in children with MPP. Based on a large number of existing randomized controlled trials, we firstly use the meta-analysis to systematically evaluate the clinical efficacy and safety of SHL oral preparations in the treatment of MPP in children.

## Materials and methods

This study followed the statement on the Preferred Reporting Items for Systematic Reviews and Meta-Analyses (PRISMA) for randomized controlled trials [20, 21]. We have registered and published the protocol of this study in PROSPERO, and the registration number is CRD42020202129.

### Eligibility criteria

This study included randomized controlled trials (RCTs) of SHL oral preparations combined with azithromycin for Children with Mycoplasma pneumoniae pneumonia (MPP), regardless of whether the blinding and allocation concealment were implemented. SHL oral preparations include SHL granules, SHL oral liquid, and so on. This study was restricted to patients age under 15 years old. After laboratory tests and imaging tests, patients have been confirming diagnosed as MPP without underlying serious diseases and other acute infectious diseases. SHL oral preparations must comply with the standards of traditional Chinese patent medicine dosage form and can be retrieved and queried on the official website of the National Medical Products Administration. After pre-statistics on the literature, SHL oral preparations only include SHL oral liquid (SHLOL) and SHL granules (SHLG). The interventions were SHL combined with azithromycin and azithromycin alone. When necessary, researchers should give basic treatments such as reducing fever, relieving cough, expectoration, and so on. The primary outcomes of this study are response rate and disappearance time of fever, disappearance time of cough, and disappearance time of pulmonary rales. The calculation mode of response rate = (number of total patients—number of invalid patients) / number of total patients. Patients with unchanged or worsening symptoms (fever, cough, pulmonary rales, and pulmonary shadows in X-ray) were considered invalid results. The secondary outcomes included average hospitalization time; inflammatory cytokines: tumor necrosis factor-α (TNF-α), interleukin-6 (IL-6), interleukin-8 (IL-8); T lymphocytes: CD3+ T lymphocytes (CD3+), CD4 + T lymphocytes (CD4+), CD8+ T lymphocytes (CD8+), CD4+ T lymphocytes / CD8+ T lymphocytes (CD4+/CD8+). For the safety of SHL, including adverse events (AEs) and the incidence of adverse rate (AR).

#### Excluded criteria

1. The types of clinical studies were Non-RCTs; 2. RCTs with missing and error data; 3. Cases, reviews, animal experiments, cell experiments, and so on; 4. SHL preparations combined with non-azithromycin antibiotics; 5. Clinical diagnosis of severe Mycoplasma pneumoniae pneumonia (SMPP), refractory Mycoplasma pneumoniae pneumonia (RMPP); 6. patients were adults or children (age≥15 years old); 7. other non-oral SHL preparations.

### Search strategy

We retrieved three Chinese databases (China National Knowledge Infrastructure, Wan Fang Data, and VIP) and five English databases (PubMed, Cochrane Library, Embase, Web of Science, and MEDLINE database) from the inception of these databases to September 2020, to include the randomized controlled trial of SHL combined with azithromycin for MPP in children. There are no language restrictions on the retrieved literature. Besides, we reviewed the relevant references of eligible literature and contacted the authors to obtain unpublished data. In addition, we also searched the clinical trial registry center. The detailed retrieval strategy was shown in S1 File.

### Literature screening and data extraction

Two researchers, (YP and YL), independently searched seven databases with the same inclusion and exclusion criteria. After the duplicate checking of the initial search and supplementary data, we screen the title, abstract, and full-text of the literature to select the RCTs that meet our inclusion criteria. In the process of review, if there are any ambiguities, it can be resolved by the third researcher (XL).

Two researchers, (YP and YL), separately extracted the data in the included RCTs. The data extraction contents mainly included: (1) Basic of included study characteristics: first author, publication time, the number of patients, course of treatment, dosage form; (2) Characteristics of subjects: age, sex, course of disease; (3) Interventions: SHL preparations (SHL granules and SHL oral liquid) combined with azithromycin and azithromycin alone; (4) Details of outcomes.

### Risk of bias assessment

Two researchers (QL and HL) used the Cochrane risk of bias tool to evaluate the included RCTs. This study mainly evaluates the bias of RCTs from seven aspects, which are random sequence generation, allocation concealment, blinding of participants and personnel, blinding of outcome assessment, incomplete outcome data, selective reporting, and other bias. Each item has three levels, which are the low-risk level, unclear risk level, and high-risk level, respectively. If there were any disagreements of evaluation, the third author (XL) settled these disagreements.

### Statistical analysis

The response rate and the incidence of adverse rate were the dichotomous data to use the risk ratio (RR) with 95% confidence intervals (95%CI). Other outcomes are continuous data to use the mean difference (MD) with 95% CI. The effect model was selected by the heterogeneity. Large heterogeneity (P < 0.1, I^2^ > 50%) use the random-effect model. When the heterogeneity is small (P ≥ 0.1, I^2^ ≤ 50%), the fixed-effect model is used. When the literature volume of the outcomes was ≥ 10, the publication bias was detected by the funnel plot (P <0.05 indicated the presence of publication bias).

To explore heterogeneity, we use the meta-regression to analyzed the potential confounders. Six regressors were considered as follows: the sample size, age, course of treatment, course of the disease, dosage form, and modes of administration. For the missing data, we use multiple imputations to supply the missing data. Besides, we also use sensitivity analysis for heterogeneity tests to further enhance the stability of the results. The Grading of Recommendations, Assessment, Development, and Evaluation (GRADE)have been used to assess the certainty of evidence in primary outcomes and safety. Use RevMan 5.3 and R language to analyze the data. Risk bias assessment has used by Cochrane Revman5.3.

## Results

### Literature review

**Fig 1:**
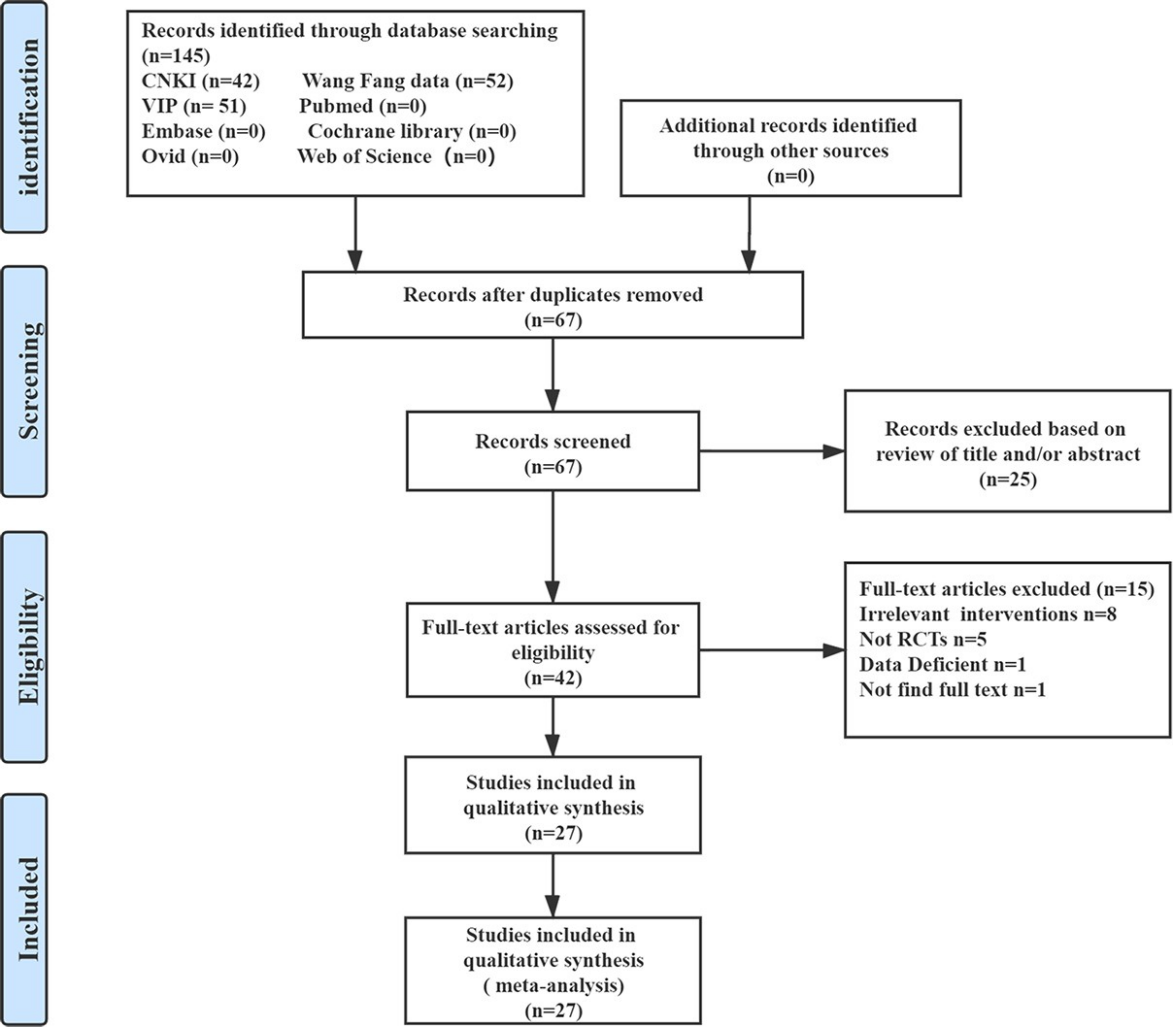
Summary of evidence search and selection.

In this meta-analysis, three Chinese databases and five English databases were systematically searched until September 2020, and we initially identified 145 potentially eligible studies. Besides, we also checked the references of relevant studies, and no additional studies that met the criteria were supplemented. After the initial screening, we excluded 78 repeated studies and reduced to 67 studies. The titles and abstracts have been screened, leaving 42 studies to be screened at full-text with same inclusion criteria. 27 proved potentially eligible after reviewing the full-text, and 15 studies were excluded. After literature screening and eligibility identification, a total of 27 studies were eventually included into this qualitative meta-analysis (Fig 1).

### Studies and characteristics

A total of 27 studies [22–48] have met the inclusion criteria, involving 2884 children (1561 males and 1323 females). The median age was 6.56 years with the range from 4 to 13 years. The median course of disease was 6.79 days with a range from 3 to 13 days. The average course of treatment was 15.73 days. The median duration of the disease was 6.79 days with a range from 3 to 13 days. The average treatment duration was 15.73 days. The interventions of the experiment group were the SHL combined with azithromycin, involving the SHL oral liquid (SHLOL) combined with azithromycin (10 studies); SHL granules (SHLG) combined with azithromycin (17 studies). The intervention of the control group was azithromycin alone (27 studies). Sequential therapy of azithromycin has been used in 11 studies, and the administration method of the remaining studies was oral therapy. 18 studies have reported adverse events. The dosage of SHL as follows: dosage of SHL oral liquid was 30-60ml/day (1-3age:30ml/day; 4-7age:60ml/day), and dosage of SHL granule was15g/day. The dose of azithromycin as follows: sequential therapy was included that intravenous injection with about 10-15mg/(kg·day) and oral therapy with 10-12mg/(kg·day), and oral therapy alone was 8–10 mg/(kg·day) (S1 Table).

### Methodological quality

We evaluated the risk of bias of included 27 studies, 9 (33.33%) studies fully reported their randomization sequence. None of the studies mentioned the distribution concealment. Only 2 (7.4%) studies reported blinding to patients and researchers, and none of the studies reported blinding to the result evaluators. All the studies showed that there was no data missing, and the risk of bias was low risk level. 11 (40.74%) studies have not selective reporting. Other bias risks were not mentioned, which were unclear risk levels (S1 and S2 Figs).

### Results of the meta-analysis

#### Primary outcomes

**Fig 2:**
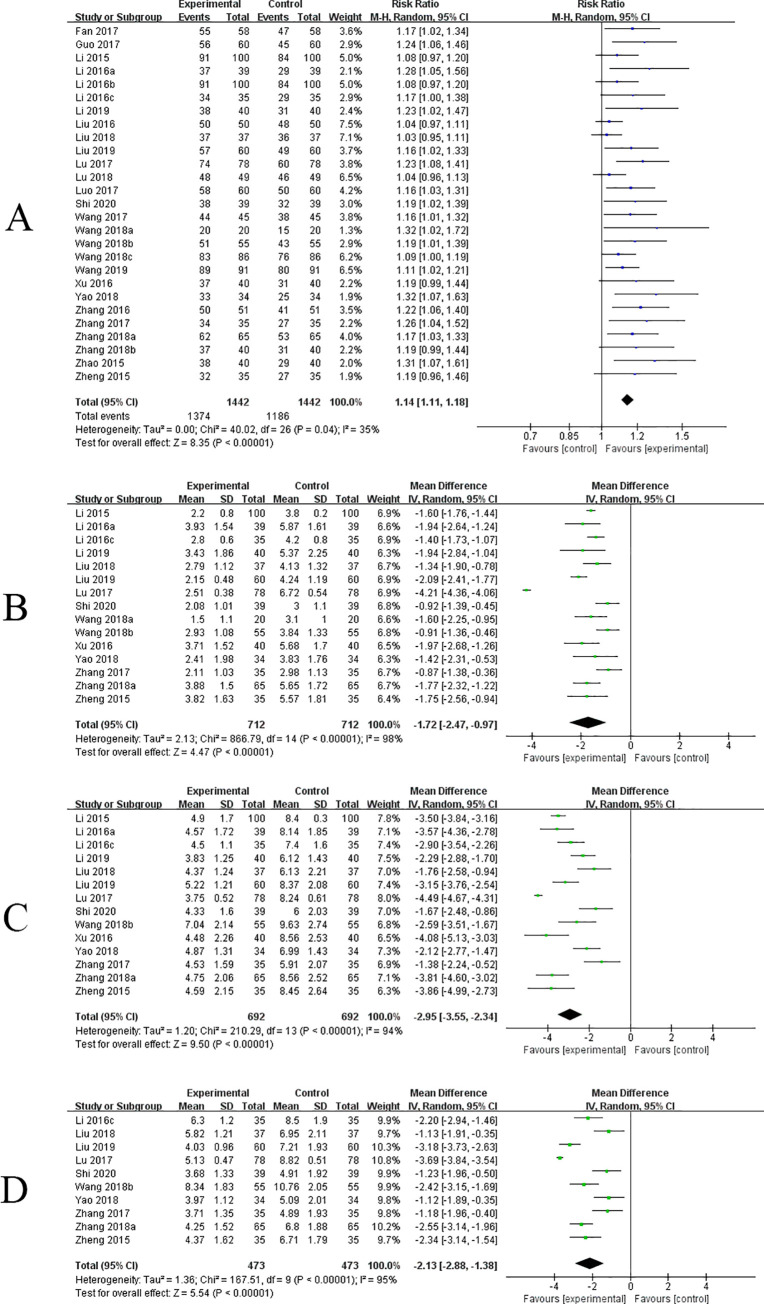
Forest plots of primary outcomes. (A) response rate; (B) disappearance time of fever; (C) disappearance time of cough; (D) disappearance time of pulmonary rales.

**Fig 3:**
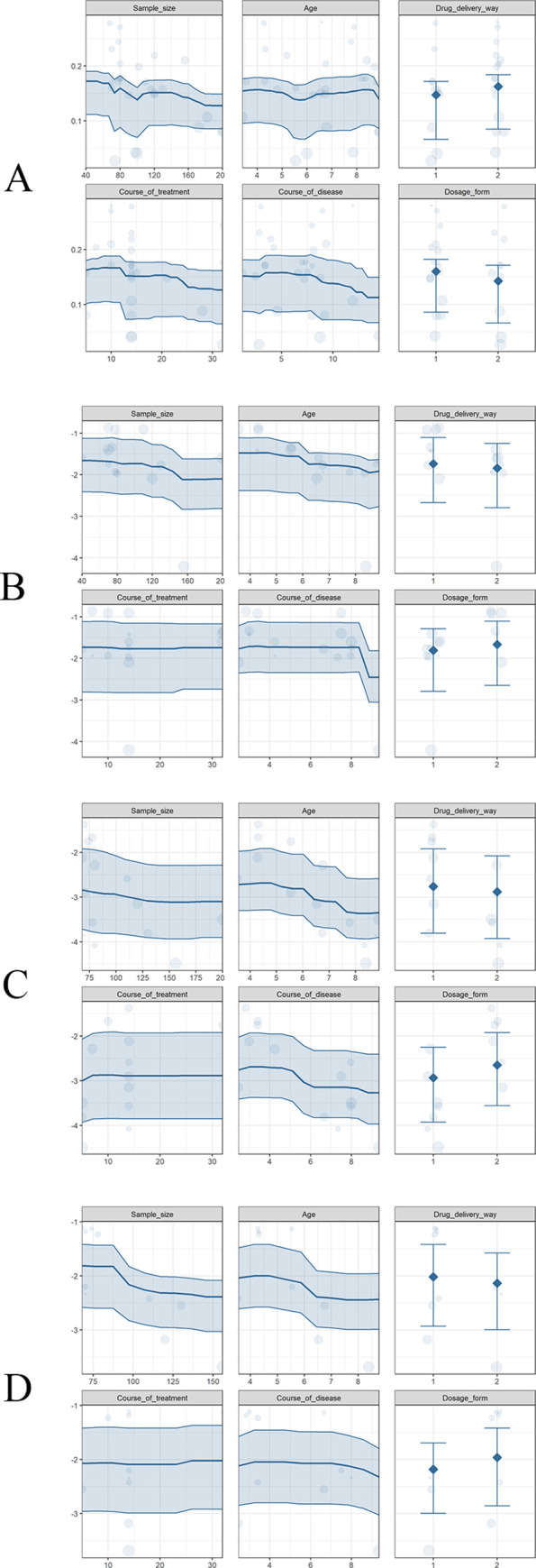
Multivariate meta-regression of primary outcomes. (A) response rate; (B) disappearance time of fever; (C) disappearance time of cough; (D) disappearance time of pulmonary rales.

*Response rate*. All 27 RCTs reported the response rate in this study, involving 2884 patients. Overall, there was a significant difference in response rate between 1374 of 1442 patients (95.28%) receiving the SHL combined with azithromycin and 1186 of 1442 patients (82.25%) receiving azithromycin alone with low certainty of evidence (RR = 1.14, 95% CI[1.11, 1.18], P<0.00001) (Fig 2A and Table 1). Multivariate meta-regression showed that, with the increase of course of the disease, the estimated value was decreased (estimate = -0.0147, P = 0.0051, 95%CI [-0.0249, -0.0044]) (Fig 3A and Table 2). The subgroup analysis for response rate has shown that the course of the disease was not the source of heterogeneity (P = 0.39, I^2^ = 0%), and did not affect the stability of the results (S2 Table). Sensitivity analysis showed that the result of the response rate was validated as relatively stable and credible (S3A Fig).

**Table 1:** Grading of recommendations, assessment, development, and evaluation of primary outcomes and safety of SHL+Azithromycin compared with azithromycin.

Outcomes	Detail of Outcomes	Studies	Sample size (T/C)	Relative effect (95%CrI)	Quality of evidence* (GRADE Assessment)
**Primary Outcomes**	Response rate	27	1442/1442	RR 1.14 (1.11, 1.18)	Low^1,5^
	Disappearance time of fever	15	712/712	MD -1.72 (-2.47, -0.97)	Low^1,2^
	Disappearance time of cough	14	692/692	MD -2.95 (- 3.55, -2.34)	Low^1,2^
	Disappearance time of pulmonary rales	10	473/473	MD -2.13 (-2.88, -1.38)	Low^1,2^
**Safety**	Adverse rate	17	939/939	MD 0.44 (0.35, 0.56)	Low^1,5^

**RR:** Odds Ratio; **MD:** Mean Difference; **95% CrI:** 95% Credible Interval.

**GRADE:** Grading of Recommendations, Assessment, Development, and Evaluation.

**Interventions:** SHL: SHL oral preparations

* Estimates for primary outcomes and safety with the Grading of Recommendations, Assessment, Development, and Evaluation Assessment: 1. downgraded because of risk of bias; 2. downgraded because of inconsistency; 3. downgraded because of indirectness; 4. downgraded because of imprecision; 5. downgraded because of publication bias.

**Table 2:** The meta-regression results of primary outcomes.

	Category of regression†	Estimate	Pval	95%CI
**Response rate**	Sample size	-0.0004	0.2071	[-0.001,0.0002]
	Age	-0.0071	0.4422	[-0.0251, 0.0109]
	Drug delivery way	0.0369	0.4143	[-0.0517, 0.1255]
	Course of treatment	-0.0028	0.1414	[-0.0065, 0.0009]
	Course of disease	-0.0147	0.0051	[-0.0249, -0.0044]**
	Dosage form	-0.077	0.1388	[-0.1790, 0.0249]
**Disappearance time of fever**	Sample size	-0.0051	0.5055	[-0.0202, 0.0100]
	Age	-0.2208	0.3684	[-0.7018, 0.2603]
	Drug delivery way	-0.5795	0.5857	[-2.6637, 1.5046]
	Course of treatment	0.0261	0.3308	[-0.0265, 0.0787]
	Course of disease	-0.0406	0.7877	[-0.3360, 0.2548]
	Dosage form	-0.5253	0.6281	[-2.6512, 1.6005]
**Disappearance time of cough**	Sample size	0.0113	0.0758	[-0.0012, 0.0238]
	Age	-0.6432	0.0007	[-1.0165, -0.2698]***
	Drug delivery way	1.1272	0.0477	[0.0112, 2.2432]*
	Course of treatment	0.0464	0.0892	[-0.0071, 0.1000]
	Course of disease	-0.331	0.0004	[-0.5148, -0.1473]***
	Dosage form	0.609	0.4229	[-2.0987, 0.8806]
**Disappearance time of pulmonary rale**	Sample size	-0.026	<0.0001	[-0.0356, -0.0165]***
	Age	-0.0765	0.5357	[-0.3184, 0.1655]
	Drug delivery way	-1.0331	0.0119	[-1.8384, -0.2278]*
	Course of treatment	-0.074	0.1061	[-0.1639, 0.0158]
	Course of disease	0.5018	0.0098	[0.1212, 0.8824]**
	Dosage form	1.9003	0.0824	[-0.2443, 4.0449]

†Category of regression were the covariate that may affect our results in this manuscript.

Pval: P value; 95%CI: 95% confidence interval

Significant codes

‘***’: Pval (0–0.001)

‘**’: Pval (0.001–0.01)

‘*’: Pval (0.01–0.05).

*Disappearance time of fever*. 15 RCTs reported the disappearance time of fever, involving 1424 patients. We found a statistically significant difference in the comparison between SHL combined with azithromycin and azithromycin alone with low certainty of evidence (MD = -1.72, 95% CI[-2.47, -0.97], P<0.00001) (Fig 2B and Table 1). Heterogeneity of different studies was considerable (P < 0.00001, I^2^ = 98%), meta-regression has been conducted to analyze the possible confounders. Multivariate meta-regression found no interactions between sample size, age, drug delivery way, course of treatment, course of disease, or dosage form and effects on disappearance time of fever, so that heterogeneity could not be completely explained (Fig 3B and Table 2). Sensitivity analysis revealed that one study [32] as a likely source of heterogeneity. After excluding this study, the results have changed (MD = -1.51, 95% CI [- 1.74, -1.28]), but it did not influence the stability of the result (S3B Fig).

*Disappearance time of cough*. 14 RCTs reported the disappearance time of cough, involving 1384 patients. Compared with azithromycin, SHL combined with azithromycin was a significant association with Disappearance time of cough, and the difference had statistical significance with low certainty of evidence (MD = -2.95, 95% CI [- 3.55, -2.34], P < 0.00001) (Fig 2C and Table 1). Heterogeneity of different studies was considerable (P < 0.00001, I^2^ = 94%), the regression results showed that the estimated value decreased with the increase of age and course of disease, and changed with the mode of administration (Fig 3C). Age (estimate = - 0.6432, P = 0.0007, 95%CI [-1.0165, -0.2698]), course of disease (estimate = -0.331, P = 0.0004, 95%CI [-0.5148, -0.1473]), and drug delivery way (estimate = 1.1272, P = 0.0477, 95%CI [0.0112, 2.2432]) may be the source of heterogeneity (Table 2). The subgroup analysis further demonstrated that age (P = 0.0002, I^2^ = 93%), course of disease (P = 0.0001, I^2^ = 93.2) and drug delivery way (P = 0.02, I^2^ = 82.3%) were the source of heterogeneity, but they did not affect the stability of the results (S2 Table). Sensitivity analysis confirmed that the result was relatively stable (S3C Fig).

*Disappearance time of pulmonary rales*. 10 RCTs reported the disappearance time of pulmonary rales, involving 946 patients. There was a statistically significant difference in the comparison between SHL combined with azithromycin and azithromycin alone with low certainty of evidence (MD = -2.13, 95% CI[-2.88, -1.38], P<0.00001) (Fig 2D and Table 1). Heterogeneity of different studies was considerable (P<0.00001, I^2^ = 95%), the regression results showed that the estimated value decreased with the increase of simple size and course of disease, and changed with the mode of administration (Fig 3D). Simple size (estimate = -0.026, P = <0.0001, 95%CI [-0.0356, -0.0165]), course of disease (estimate = 0.5018, P = 0.0098, 95%CI [0.1212, 0.8824]), and drug delivery way (estimate = -1.0331, P = 0.0119, 95%CI [-1.8384, -0.2278]) may be the source of heterogeneity (Table 2). The subgroup analysis further demonstrated that age (P = 0.0004, I2 = 92.1%), course of disease (P = 0.16, I^2^ = 49.7%) and drug delivery way (P = 0.00004, I^2^ = 92.1%) were the source of heterogeneity, but they did not affect the stability of the results (S2 Table). Sensitivity analysis revealed that one study (Lu 2017) has yielded the heterogeneity. After excluding this study, the result have changed (MD = -1.95, 95% CI[-2.47, -1.44]), but it did not influence the stability of the result (S3D Fig).

#### Secondary outcomes

*Average hospitalization time*. 7 RCTs reported the Average hospitalization time, and there was a statistically significant difference in the comparison between SHL combined with azithromycin and azithromycin alone (MD = -4.19, 95% CI[-5.03, -3.34], P<0.00001) (S4 Fig).

*T lymphocytes*. 9 RCTs, 10 RCTs, 5RCTs and 7RCTs reported CD3+ T-lymphocytes, CD4+ T-lymphocytes, CD8+ T-lymphocytes and CD4+/CD8+, respectively. For CD3+ T-lymphocytes (MD = 6.68, 95% CI[5.29, 8.06], P<0.00001), CD4+ T-lymphocytes (MD = 4.78, 95% CI[3.76, 5.81], P<0.00001), CD8+ T-lymphocytes (MD = -4.52, 95% CI[-5.28, -3.77], P<0.00001) and CD4/CD8 (MD = 0.31, 95% CI[0.14, 0.47], P = 0.0002), there were statistically significant difference in the comparison between SHL combined with azithromycin and azithromycin alone (S5 Fig).

*Inflammatory cytokine*. 10 RCTs, 6 RCTs and 7RCTs reported IL-6, IL-8 and TNF-α, respectively. For IL-6 (MD = -8.35, 95% CI[-12.31, -4.4], P<0.0001), IL-8 (MD = -4.49, 95% CI[-5.12, -3.87], P<0.00001) and TNF-α (MD = -9.09, 95% CI[-10.91, -7.27], P<0.00001), there were statistically significant difference in the comparison between SHL combined with azithromycin and azithromycin alone (S6 Fig).

*Safety*. AEs were reported in 18 studies. Details of all AEs were as follows: 18 trials of SHL combined with azithromycin were reported 91 AEs (SHLG combined with azithromycin: 10 trials, 58 AEs; SHLOL combined with azithromycin: 8 trials, 33 AEs). The control group was azithromycin (18 trials, 201 AEs). Xu 2016 only reported the total number of AEs (SHLG combined with azithromycin: 5 AEs; azithromycin: 16 AEs), and there was no detailed description. The AEs of interventions were as follows: 1. SHLG combined with azithromycin: abdominal pain and diarrhea (8 trials, 21 AEs), nausea and vomiting (9 trials, 16 AEs), rash (7 trials, 12 AEs), abnormal liver and kidney (2 trials, 4 AEs); 2. SHLOL combined with azithromycin: abdominal pain and diarrhea (7 trials, 11 AEs), nausea and vomiting (5 trials, 11 AEs), rash (5 trials, 7 AEs), injection local site pain (1 trial, 2 AEs), unreported gastrointestinal reaction (1 trial, 2 AEs); 3. azithromycin alone: abdominal pain and diarrhea (14 trials, 58 AEs), nausea and vomiting (15 trials, 58 AEs), rash (11 trials, 31 AEs), hepatorenal abnormalities (4 trials, 16 AEs), dyspepsia (1 trials, 4 AEs), unreported gastrointestinal reactions (1 trial, 3 AEs), injection local pain (1 trial, 2 AEs), cholestasis jaundice (1 trial, 2 AEs), somnolence (1 trial, 5 AEs), pericarditis (1 trials, 4 AEs), aseptic meningitis (1 trial, 2 AEs) (S1 Table).

**Fig 4:**
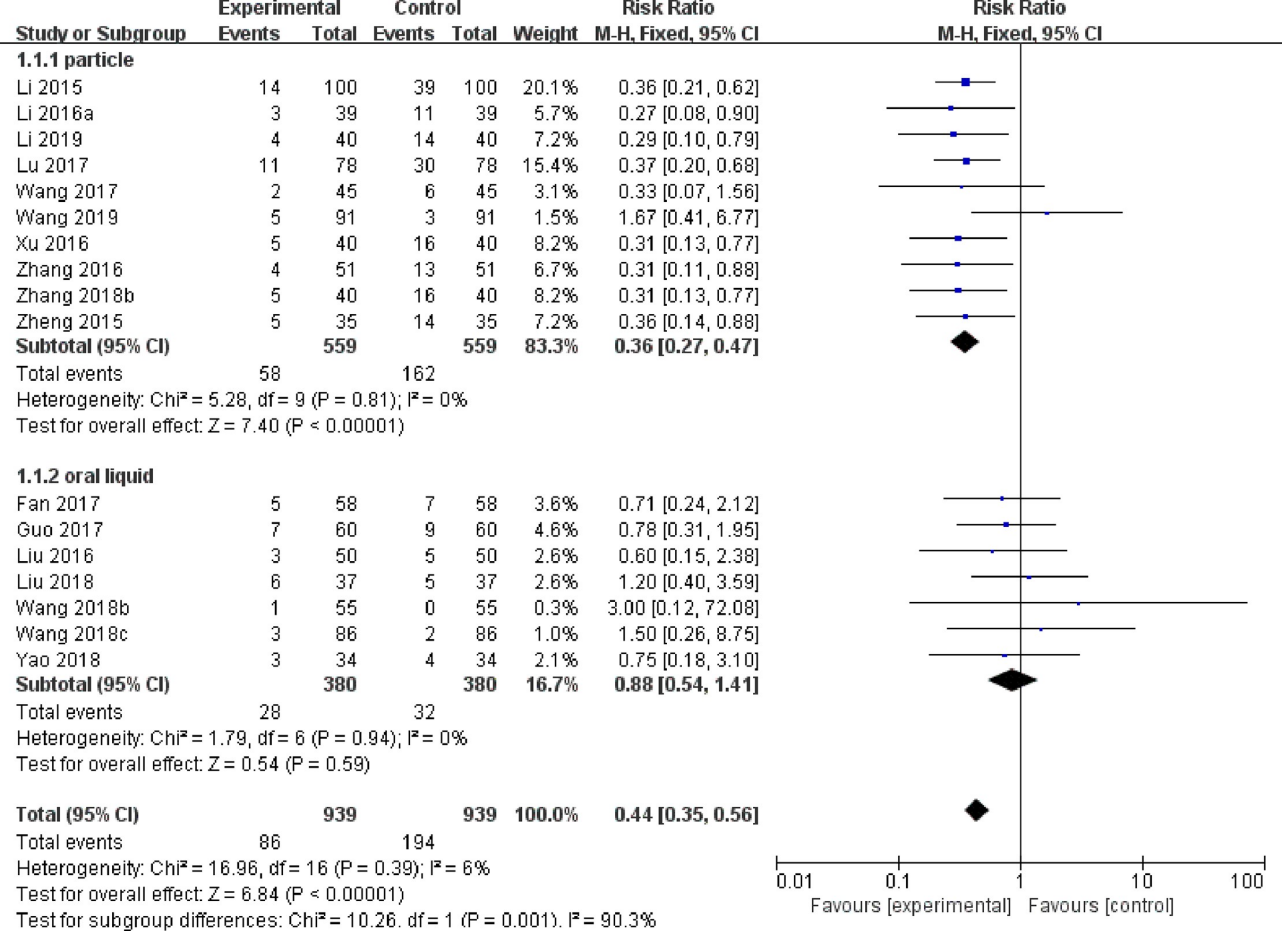
Forest Plots of adverse rate.

Of 17 RCTs reported the incidence of AR. There was a statistically significant difference between SHL combined with azithromycin and azithromycin alone with low certainty of evidence (MD = 0.44, 95% CI[0.35, 0.56], P<0.00001) (Fig 4 and Table 1). The subgroup analysis demonstrated that the mode of administration (P = 0.001, I^2^ = 90.3%) was the source of heterogeneity. Compared with azithromycin, SHLG combined with azithromycin was a statistically significant difference (MD = 0.36, 95% CI [0.27, 0.47], P < 0.00001), but SHLOL combined with azithromycin was not a significant difference (MD = 0.88, 95% CI[0.54, 1.41], P = 0.59) (Fig 4).

### Publication bias

**Fig 5:**
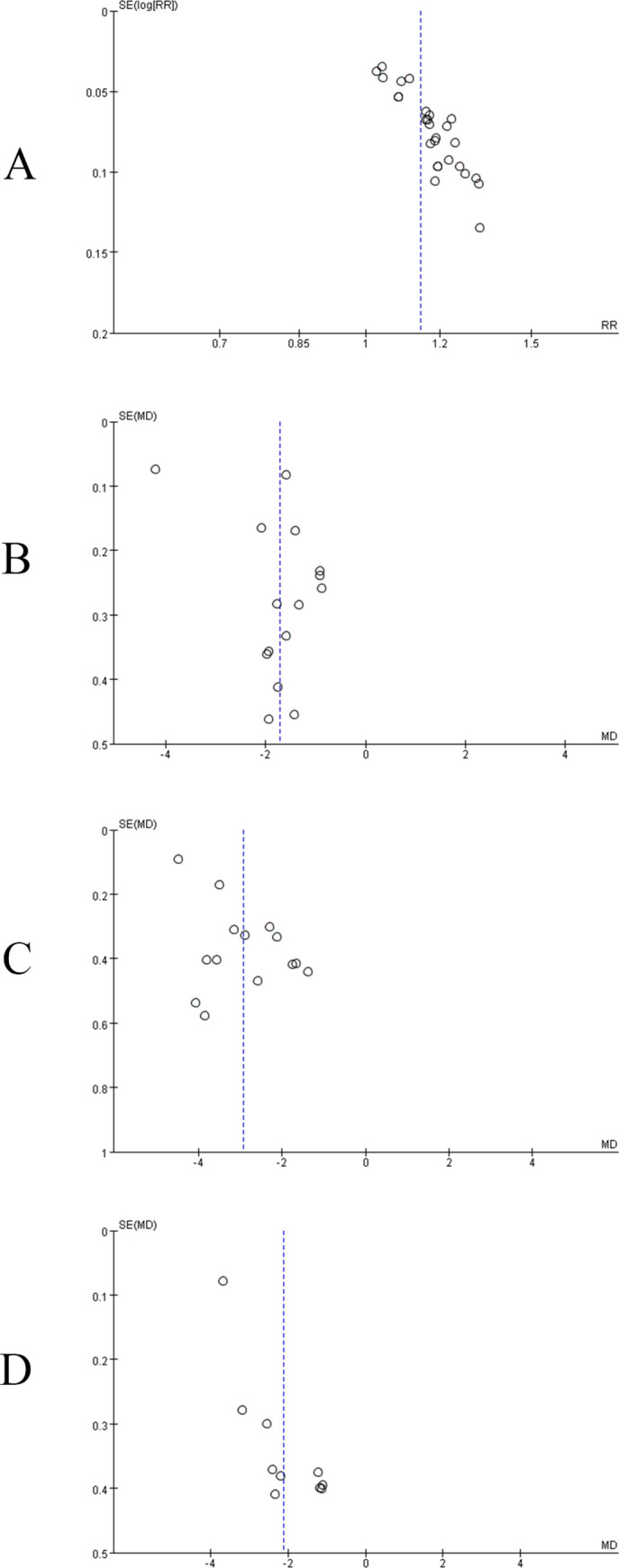
The funnel plot of primary outcomes. A: response rate; B: disappearance time of fever; C: disappearance time of cough; D: disappearance time of pulmonary rales.

We draw the funnel plots of the primary outcomes to assess publication bias. The distributions of response rate, disappearance time of fever, disappearance time of cough, and disappearance time of pulmonary rales were asymmetric that might be publication bias (Fig 5).

## Discussion

### Summary of evidence

This study systematically evaluated the efficacy and safety of SHL combined with azithromycin in the treatment of MPP in children. A total of 27 studies were included, involving 2884 children. SHL oral preparations include SHLOL (10 trials) and SHLG (17 trials). Most studies reported primary outcomes and safety, only a few studies reported secondary outcomes. Most of the included studies have a moderate risk of bias.

For primary outcomes (response rate, disappearance time of fever, disappearance time of cough, and disappearance time of pulmonary rales), We found that SHL combined with azithromycin was significantly better than azithromycin alone with low certainty of evidence. Because of the potential confounding factors, meta-regression has been used to find the source of heterogeneity that may affect the primary outcomes, and the subgroup analysis has been selected to explore the heterogeneity which may affect the stability of the results. The results of meta-regression showed that the course of disease was the heterogeneity of response rate; age, course of disease, and mode of administration were the heterogeneity of disappearance time of cough; sample size, course of disease, and mode of administration were the heterogeneity of disappearance time of pulmonary rales. Nevertheless, these sources of heterogeneity did not significantly affect the stability of the results. The sensitivity analysis indicated the results were still robust. We found that Lu 2017 [32] was the main heterogeneity, but excluding it does not affect the stability of the results. We have to believe that the results of the primary outcomes were stable and credible. We believed that the results of the primary outcomes were stable based on the meta-regression, subgroup analysis and sensitivity analysis, and the low certainty evidence of primary outcomes suggested that there may be important difference between SHL combined with azithromycin and azithromycin alone.

There are a large number of secondary outcomes, including average hospitalization time, T lymphocytes (CD3+, CD4+, CD8+, and CD4+/CD8+), and inflammatory cytokine (IL-6, IL-8, and TNF-α). Meta-analysis results of all secondary outcomes showed that SHL combined with azithromycin was better than azithromycin.

In safety, 66.67% of the studies (18 trials) reported the AEs, and 62.96% of the studies (17 trials) reported the incidence of ADRs. Azithromycin alone reported 201 AEs, while SHL combined with azithromycin have reports 91 AEs (SHLG: 58 AEs; SHLOL: 33AEs). The AEs of azithromycin alone was twice as many as that of SHL combined with azithromycin. Of all the reported AEs, nausea, and vomiting, abdominal pain, diarrhea and rash were the most frequent. Azithromycin alone reported hepatorenal abnormalities, pericarditis, cholestatic jaundice, and aseptic meningitis, no several AEs were discovered in SHL combined with azithromycin. The incidence of ADRs showed that the difference between SHL preparation combined with azithromycin and azithromycin was statistically significant with low certainty of evidence. Subgroup analysis showed that SHLG combined with azithromycin was significantly better than azithromycin alone, but there was no significant difference between SHLOL combined with azithromycin and azithromycin alone. More attention should be paid to the safety differences of different dosage forms in the future. Overall, the safety of oral preparations with low certainty evidence is that results support SHL combined with azithromycin may be safety with no serious AEs.

SHL oral preparations with a long history of clinical applications for pneumonia were the TCPMs approved by the State Drug Administration, and its clinical efficacy was relatively stable and safe. SHL oral preparations are composed of Lonicerae Japonicae Flos, Scutellariae radix, and Forsythia Fructus, and have a variety of active compounds with hydrophilic and lipophilic components [49]. Related pharmacological studies have shown that SHL granules can reduce the levels of TNF- α and IL-6 through NF- κ B, to effectively improve pulmonary inflammation and restore lung function [50]. In addition, it can also inhibit repeated MP infection by affecting the protein expression of platelet-derived growth factor, and prevent pulmonary interstitial fibrosis caused by the aggravation of pneumonia [51]. Metabonomic studies confirmed that SHL can improve the fever caused by pneumonia through five kinds of potential perturbed metabolic pathways to play the role of anti-fever [52]. According to the results of network pharmacology of SHL for MPP, we found that SHL can exert its efficacy and explain its complex pharmacological mechanism through the "multi-component, multi-target, and multi-pathway", which also provides a guarantee and basis for the safety of SHL [53].

### Strengths and limitations

In China, SHL oral preparations have been widely used by clinicians in the clinical treatment of MPP in children. This study has evaluated, for the first time, the efficacy and safety of SHL oral preparations combined with azithromycin in the treatment of children with MPP. We have searched nine Chinese and English databases, used more detailed and accurate retrieval strategies, strictly controlled the inclusion and exclusion criteria, and minimized the possible risk of publication bias. Due to characteristics of the disease and clinical medicine, six covariates that may cause heterogeneity was analyzed by meta-regression to reduce the possible confounding factors. Besides, we also use subgroup analysis and sensitivity analysis to fully explore and explain the sources of heterogeneity that may affect the stability of the results.

This study has the following limitations. None of the included studies reported allocation concealment, and only 7.4% of studies (2 trials) mentioned blind methods, which may affect the internal authenticity of the results and cause risk of bias. All the included studies were Chinese, and the subjects were Asian. SHL oral preparations were superior to azithromycin alone in the incidence of ADRs, but its safety was not conclusive. All the included studies not mentioned follow-up. Previous studies have reported that traditional Chinese medicine may reduce the macrolide resistance [19]. However, this study did not show whether SHL oral preparations can improve the ADRs of azithromycin. Included studies have lacked attention to some important outcomes that control rate, recurrence, economic status, quality of life, and we hope that future research can pay attention to these outcomes. Information related to research and disease (such as comorbidities, follow-up time, number of people lost to follow-up and reasons) has not been reported in RCTs, which may lead to a lack of data during the follow-up period and the neglect of complications. The primary outcomes have some publication bias.

## Conclusions

In conclusion, this study confirmed that SHL oral preparations combined with azithromycin most likely to more effective than azithromycin alone for MPP in Asian children. For Safety, SHL oral preparations may be safety with no serious adverse events, and the safety needs further verification. Because of the low certainty of evidence for primary outcomes and safety, we must be cautious in clinical applications. Due to these limitations, more high-quality randomized controlled trials will be conducted to further confirm.

## Supporting information

S1 Checklist PRISMA checklist. (DOC)

S1 Fig Risk of bias of included studies. (TIF)

S2 Fig Quality assessment for each included study. (TIF)

S3 Fig Sensitivity analysis of primary outcomes. (A) response rate; (B) disappearance time of fever; (C) disappearance time of cough; (D) disappearance time of pulmonary rales.
(TIF)

S4 Fig Average hospitalization time. (TIF)

S5 Fig T lymphocytes. (A) CD3+ T lymphocytes (CD3+); (B) CD4 + T lymphocytes (CD4+); (C) CD8+ T lymphocytes (CD8+); (D) CD4+ T lymphocytes / CD8+ T lymphocytes (CD4+/CD8+).
(TIF)

S6 Fig Inflammatory cytokine. (A) Interleukin-6 (IL-6); (B) Interleukin-8 (IL-8); (C) Tumor necrosis factor-α (TNF-α).
(TIF)

S1 Table Characteristics of included studies. Groups: T: Treatment group; C: Control group; Interventions: SHL: Shuanghuanlian (oral liquid/ granules). Outcome measurements: 1: response rate; 2: disappearance time of fever; 3: disappearance time of cough; 4: disappearance time of pulmonary rales; 5: average hospitalization time; 6: CD3+ T lymphocytes (CD3+); 7: CD4 + T lymphocytes (CD4+); 8: CD8+ T lymphocytes (CD8+); 9: CD4+ T lymphocytes / CD8+ T lymphocytes (CD4+/CD8+); 10: interleukin-6 (IL-6); 11: interleukin-8 (IL-8); 12: tumor necrosis factor-α (TNF-α); 13: adverse events.
(DOCX)

S2 Table The results of subgroup analysis. (DOCX)

S3 Table Adverse events. Groups: T: Treatment group; C: Control group; Interventions: SHL: Shuanghuanlian (oral liquid/ granules).
(DOCX)

S1 File The detailed search strategy. (DOCX)

S1 Dataset Datasets of all outcomes and safety. (DOCX)
