# Supplementary figures and images for: Shuanghuanglian oral preparations combined with azithromycin for treatment of Mycoplasma pneumoniae pneumonia in Asian children: A systematic review and meta-analysis of randomized controlled trials
Source: PLoS One. 2021 Jul 13;16(7):e0254405. doi: 10.1371/journal.pone.0254405 (PMC8277054; doi:10.1371/journal.pone.0254405)

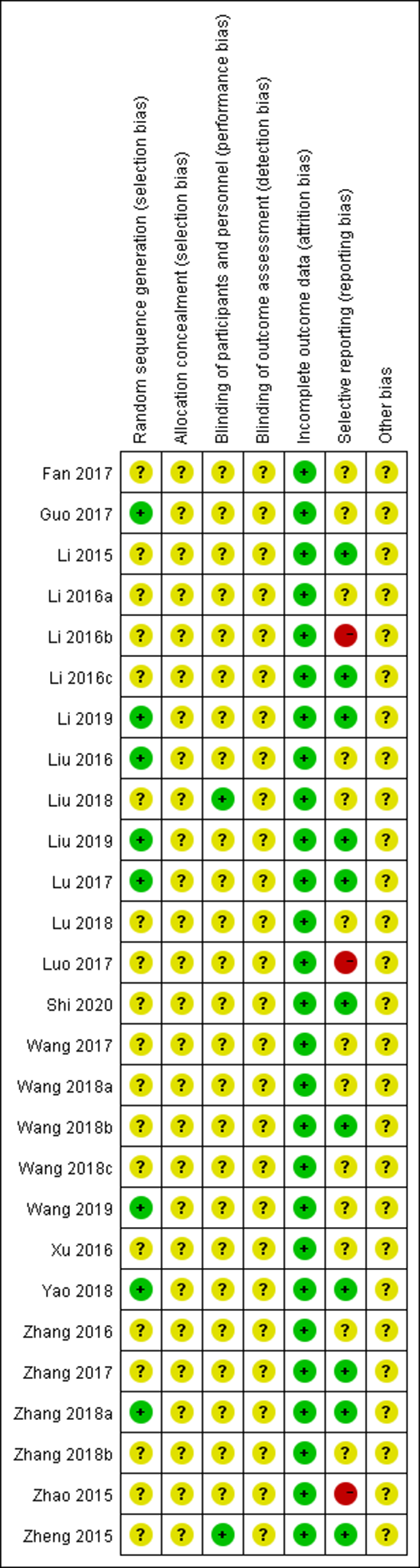

Supplement: S2 Fig — (TIF) [file pone.0254405.s003.tif]

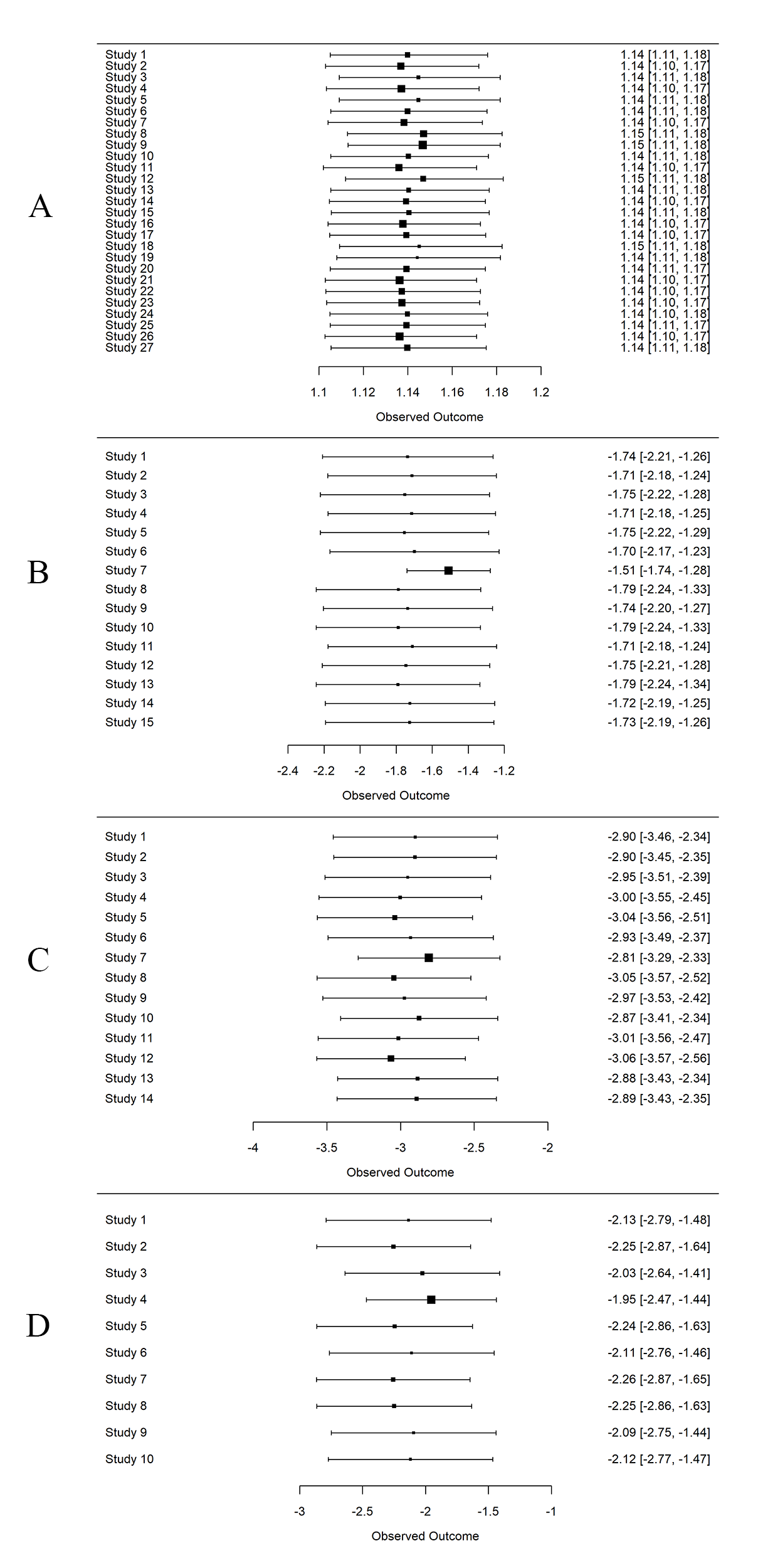

Supplement: S3 Fig — (A) response rate; (B) disappearance time of fever; (C) disappearance time of cough; (D) disappearance time of pulmonary rales. (TIF) [file pone.0254405.s004.tif]

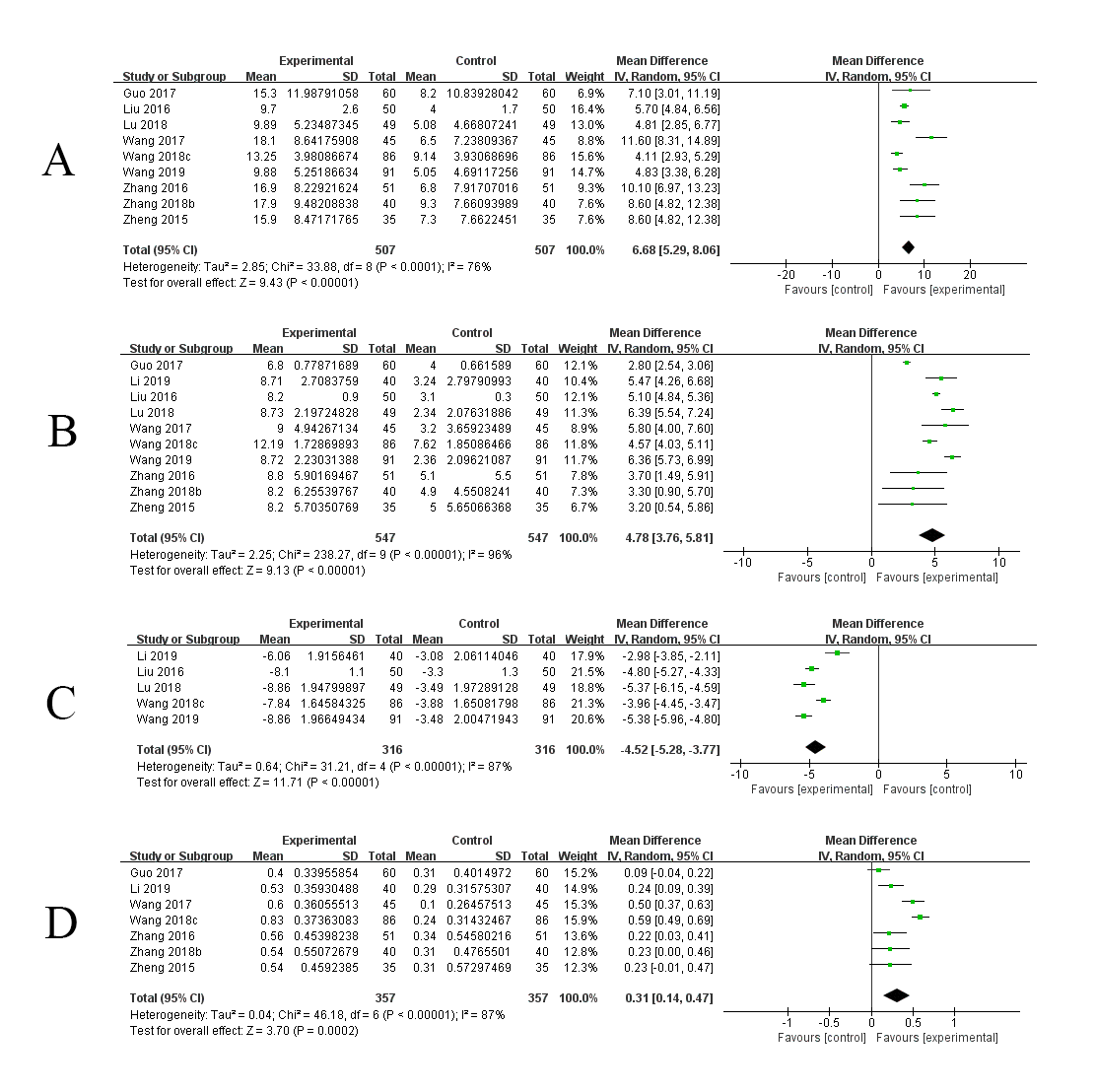

Supplement: S5 Fig — (A) CD3+ T lymphocytes (CD3+); (B) CD4 + T lymphocytes (CD4+); (C) CD8+ T lymphocytes (CD8+); (D) CD4+ T lymphocytes / CD8+ T lymphocytes (CD4+/CD8+). (TIF) [file pone.0254405.s006.tif]

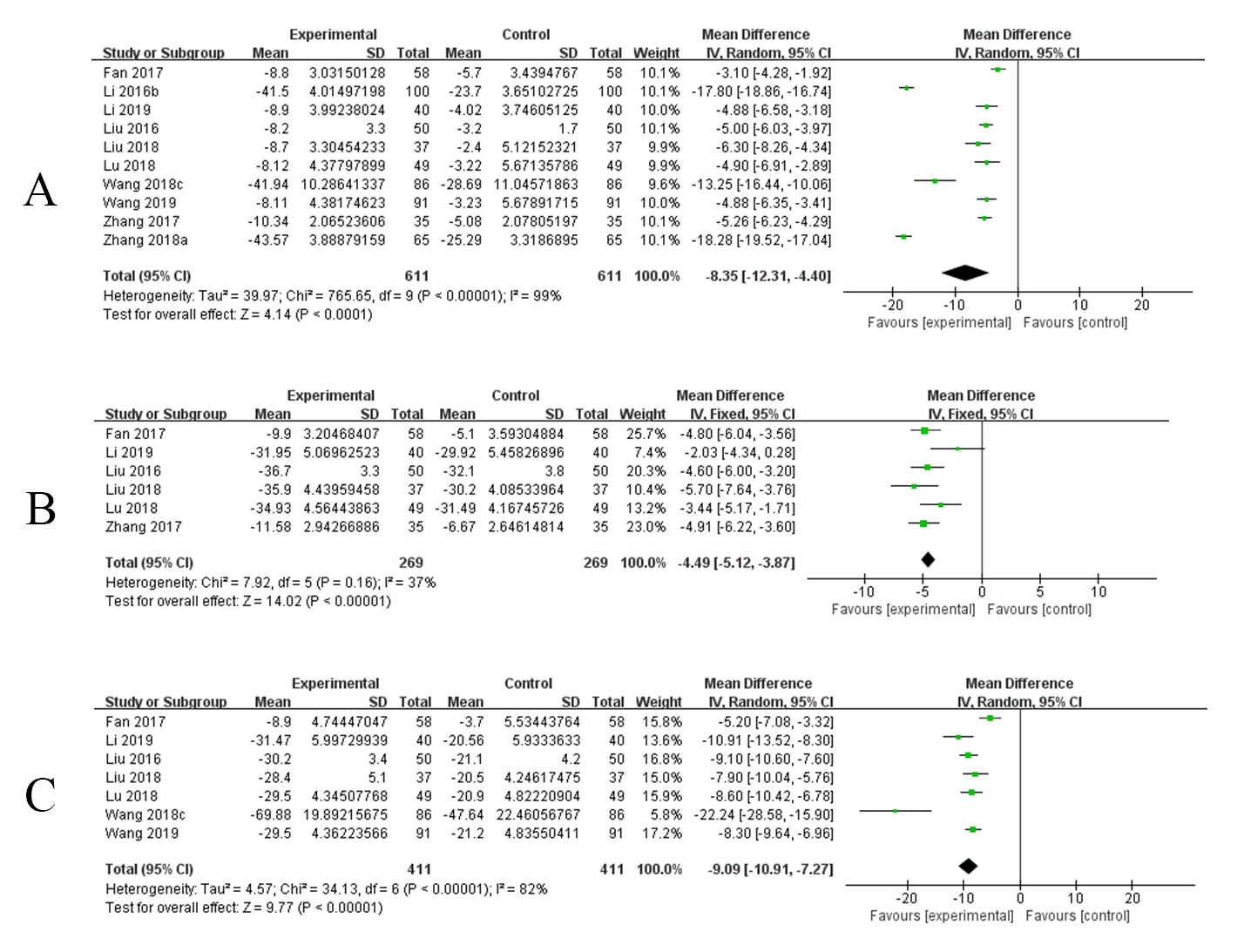

Supplement: S6 Fig — (A) Interleukin-6 (IL-6); (B) Interleukin-8 (IL-8); (C) Tumor necrosis factor-α (TNF-α). (TIF) [file pone.0254405.s007.tif]
