# Supplementary material for: Shuanghuanglian oral preparations combined with azithromycin for treatment of Mycoplasma pneumoniae pneumonia in Asian children: A systematic review and meta-analysis of randomized controlled trials
Source: PLoS One. 2021 Jul 13;16(7):e0254405. doi: 10.1371/journal.pone.0254405 (PMC8277054; doi:10.1371/journal.pone.0254405)
Supplement: S1 Table — Groups: T: Treatment group; C: Control group; Interventions: SHL: Shuanghuanlian (oral liquid/ granules). Outcome measurements: 1: response rate; 2: disappearance time of fever; 3: disappearance time of cough; 4: disappearance time of pulmonary rales; 5: average hospitalization time; 6: CD3+ T lymphocytes (CD3+); 7: CD4 + T lymphocytes (CD4+); 8: CD8+ T lymphocytes (CD8+); 9: CD4+ T lymphocytes / CD8+ T lymphocytes (CD4+/CD8+); 10: interleukin-6 (IL-6); 11: interleukin-8 (IL-8); 12: tumor necrosis factor-α (TNF-α); 13: adverse events. (DOCX) [file pone.0254405.s008.docx]

# S1 Table. Characteristics of included studies

| **Study** | **Interventions（T/C）** | | **Dosage (T/C)** | | **Sample Size （T/C）** | | **Male （T/C）** | | **Female （T/C）** | | **Age （T/C）** | | **Course of disease （T/C）** | | **Course of treatment (days)** | **Outcomes** |
| --- | --- | --- | --- | --- | --- | --- | --- | --- | --- | --- | --- | --- | --- | --- | --- | --- |
| Fan 2017 | SHL oral liquid + Azithromycin | Azithromycin | Shuanghuanglian oral liquid (1-3age:10ml,po,tid; 4-7age:20ml,po,tid)+ azithromycin (sequential therapy:15mg/(kg·d),ivgtt,qd and 10~12mg/(kg·d),po,qd) | Azithromycin (sequential therapy:15mg/(kg·d),ivgtt,qd and 10~12mg/(kg·d),po,qd) | 58 | 58 | 33 | 35 | 25 | 23 | 4.9±1.4 | 4.7±1.2 | 1.21±0.22 | 1.18±0.21 | 15 | 1 10 11 12 13 |
| Guo 2017 | SHL oral liquid + Azithromycin | Azithromycin | SHL oral liquid (1-3age:10ml,po,tid; 4-7age:20ml,po,tid)+ Azithromycin (8mg/(kg·d),po,qd) | Azithromycin (8mg/(kg·d),po,qd) | 60 | 60 | 35 | 37 | 25 | 23 | 5.2±0.8 | 4.8±1 | 3.8±0.5 | 4.1±0.6 | 14 | 1 6 7 9 13 |
| Li 2015 | SHL granules + Azithromycin | Azithromycin | SHL granules (5g,po,tid) + Azithromycin (10mg/(kg·d),po,qd) | Azithromycin (10mg/(kg·d),po,qd) | 100 | 100 | 96 |  | 104 |  | 8.9±0.2 | 8.9±0.2 | 8.0±1.6 | 8.0±1.6 | - | 1 2 3 5 13 |
| Li 2016a | SHL granules + Azithromycin | Azithromycin | SHL granules (5g,po,tid) + Azithromycin (10mg/(kg·d),po,qd) | Azithromycin (10mg/(kg·d),po,qd) | 39 | 39 | 21 | 21 | 18 | 18 | 7.78±1.19 | 7.78±1.19 | 7.96±1.64 | 7.96±1.64 | - | 1 2 3 13 |
| Li 2016b | SHL granules + Azithromycin | Azithromycin | SHL granules (5g,po,tid) + Azithromycin (10mg/(kg·d),po,qd) | Azithromycin (10mg/(kg·d),po,qd) | 100 | 100 | 96 |  | 104 |  | 8.9±0.2 | 8.9±0.2 | 9.2±1.6 | 9.2±1.6 | - | 1 10 |
| Li 2016c | SHL granules + Azithromycin | Azithromycin | SHL granules (5g,po,tid) + Azithromycin (10mg/(kg·d),po,qd) | Azithromycin (10mg/(kg·d),po,qd) | 35 | 35 | 18 | 19 | 17 | 16 | 5.4±2.1 | 5.7±2.3 | 1-15 | 1-13 | - | 1 2 3 4 |
| Li 2019 | SHL granules + Azithromycin | Azithromycin | SHL granules (5g,po,tid) + Azithromycin (10mg/(kg·d),po,qd) | Azithromycin (10mg/(kg·d),po,qd) | 40 | 40 | 19 | 23 | 21 | 17 | 6.83±2.91 | 6.68±2.74 | 4.18±1.74 | 4.32±1.65 | 7 | 1 2 3 5 7 8 9 10 11 12 13 |
| Liu 2016 | SHL oral liquid + Azithromycin | Azithromycin | SHL oral liquid (1-3age:10ml,po,tid; 4-7age:20ml,po,bid or tid)+ Azithromycin (sequential therapy:10mg/(kg·d),ivgtt,qd and 10mg/(kg·d),po,qd) | Azithromycin (sequential therapy:10mg/(kg·d),ivgtt,qd and 10mg/(kg·d),po,qd) | 50 | 50 | 28 | 27 | 22 | 23 | 5.9±1.3 | 6.1±1.4 | - | - | 14 | 1 6 7 8 10 11 12 13 |
| Liu 2018 | SHL oral liquid + Azithromycin | Azithromycin | SHL oral liquid (1-3age:10ml,po,tid; 4-10age:20ml,po,tid)+ Azithromycin (sequential therapy:1-2mg/ml,ivgtt,qd and 10mg/(kg·d),po,qd) | Azithromycin (sequential therapy:1-2mg/ml,ivgtt,qd and 10mg/(kg·d),po,qd) | 37 | 37 | 15 | 14 | 22 | 23 | 5.4±1.2 | 5.7±1.1 | - | - | 32 | 1 2 3 4 10 11 12 13 |
| Liu 2019 | SHL oral liquid + Azithromycin | Azithromycin | SHL oral liquid (1-3age:10ml,po,tid; 4-7age:20ml,po,tid)+ Azithromycin (sequential therapy:1mg/(kg·d),ivgtt,qd and 1mg/(kg·d),po,qd) | Azithromycin (sequential therapy:1mg/(kg·d),ivgtt,qd and 1mg/(kg·d),po,qd) | 60 | 60 | 34 | 38 | 26 | 22 | 6.58±2.72 | 6.48±2.25 | 2.51±0.94 | 2.43±0.89 | 14 | 1 2 3 4 5 |
| Lu 2017 | SHL granules + Azithromycin | Azithromycin | SHL granules (5g,po,tid) + Azithromycin (10mg/(kg·d),po,qd) | Azithromycin (10mg/(kg·d),po,qd) | 78 | 78 | 42 | 43 | 36 | 35 | 8.5±2.4 | 8.3±2.6 | 9.2±2.1 | 9.5±2 | - | 1 2 3 4 5 13 |
| Lu 2018 | SHL granules + Azithromycin | Azithromycin | SHL granules (5g,po,tid) + Azithromycin (10mg/(kg·d),po,qd) | Azithromycin (10mg/(kg·d),po,qd) | 49 | 49 | 24 | 22 | 25 | 27 | 8.12±4.07 | 7.62±4.12 | 7-21 | 6-24 | - | 1 6 7 8 10 11 12 |
| Luo 2017 | SHL granules + Azithromycin | Azithromycin | SHL granules (5g,po,tid) + Azithromycin (10mg/(kg·d),po,qd) | Azithromycin (10mg/(kg·d),po,qd) | 60 | 60 | 33 | 31 | 27 | 29 | 6.58±2.5 | 6.87±2.32 | 8.68±1.63 | 8.85±1.76 | 21 | 1 |
| Shi 2020 | SHL oral liquid + Azithromycin | Azithromycin | SHL oral liquid (1-3age:10ml,po,tid; 4-7age:20ml,po,tid)+ Azithromycin (sequential therapy:10mg/(kg·d),ivgtt,qd and 10mg/(kg·d),po,qd) | Azithromycin (sequential therapy:10mg/(kg·d),ivgtt,qd and 10mg/(kg·d),po,qd) | 39 | 39 | 21 | 20 | 18 | 19 | 4.33±1.15 | 4.28±1.09 | 3.28±0.61 | 3.53±0.59 | 10 | 1 2 3 4 |
| Wang 2017 | SHL granules + Azithromycin | Azithromycin | SHL granules (5g,po,tid) + Azithromycin (10mg/(kg·d),po,qd) | Azithromycin (10mg/(kg·d),po,qd) | 45 | 45 | 25 | 26 | 20 | 19 | 6.22±1.29 | 6.27±1.23 | 7.22±1.03 | 7.2±1.04 | 14 | 1 6 7 9 13 |
| Wang 2018a | SHL granules + Azithromycin | Azithromycin | SHL granules (5g,po,tid) + Azithromycin (10mg/(kg·d),po,qd) | Azithromycin (10mg/(kg·d),po,qd) | 20 | 20 | 12 | 11 | 8 | 9 | 6.7±0.15 | 6.5±0.8 | - | - | - | 1 2 5 |
| Wang 2018b | SHL oral liquid + Azithromycin | Azithromycin | SHL oral liquid (1-3age:10ml,po,tid; 4-7age:20ml,po,tid)+ Azithromycin (sequential therapy:10mg/(kg·d),ivgtt,qd and 10mg/(kg·d),po,qd) | Azithromycin (sequential therapy:10mg/(kg·d),ivgtt,qd and 10mg/(kg·d),po,qd) | 55 | 55 | 35 | 33 | 20 | 22 | 3.73±1.38 | 3.38±1.15 | - | - | 14 | 1 2 3 4 13 |
| Wang 2018c | SHL oral liquid + Azithromycin | Azithromycin | SHL oral liquid (20ml,po,tid)+ Azithromycin (sequential therapy:10mg/(kg·d),ivgtt,qd and 10mg/(kg·d),po,qd) | Azithromycin (sequential therapy:10mg/(kg·d),ivgtt,qd and 10mg/(kg·d),po,qd) | 86 | 86 | 45 | 47 | 41 | 39 | 6.73±1.86 | 6.82±1.77 | 4.67±0.57 | 4.52±0.62 | 28 | 1 6 7 8 9 10 12 13 |
| Wang 2019 | SHL granules + Azithromycin | Azithromycin | SHL granules (1-3 age:5g,po,tid; ≥4 age:10g,po,tid) + Azithromycin (12mg/(kg·d),po,qd) | Azithromycin (12mg/(kg·d),po,qd) | 91 | 91 | 48 | 47 | 43 | 44 | 3.3±0.7 | 3.5±0.8 | 12.6±2.4 | 11.3±2.2 | 14 | 1 6 7 8 10 12 13 |
| Xu 2016 | SHL granules + Azithromycin | Azithromycin | SHL granules (5g,po,tid) + Azithromycin (8mg/(kg·d),po,qd) | Azithromycin (8mg/(kg·d),po,qd) | 40 | 40 | 21 | 25 | 19 | 15 | 7.9±2.1 | 8.8±2.2 | 6.8±2.2 | 7.9±2.1 | - | 1 2 3 5 13 |
| Yao 2018 | SHL oral liquid + Azithromycin | Azithromycin | SHL oral liquid (1-3age:10ml,po,tid; 4-7age:20ml,po,tid)+ Azithromycin (sequential therapy:15mg/(kg·d),ivgtt,qd and 10-12mg/(kg·d),po,qd) | Azithromycin (sequential therapy:15mg/(kg·d),ivgtt,qd and 10-12mg/(kg·d),po,qd) | 34 | 34 | 20 | 18 | 14 | 16 | 4.57±1.45 | 3.98±1.63 | 2.75±1.42 | 3.23±1.34 | 14 | 1 2 3 4 13 |
| Zhang 2016 | SHL granules + Azithromycin | Azithromycin | SHL granules (5g,po,tid) + Azithromycin (8mg/(kg·d),po,qd) | Azithromycin (8mg/(kg·d),po,qd) | 51 | 51 | 30 | 29 | 21 | 22 | 8.65±2.35 | 8.79±2.41 | 8.25±2.05 | 8.02±2.11 | - | 1 6 7 9 13 |
| Zhang 2017 | SHL oral liquid + Azithromycin | Azithromycin | SHL oral liquid (1-3age:10ml,po,tid; 4-7age:20ml,po,tid)+ Azithromycin (sequential therapy:10mg/(kg·d),ivgtt,qd and 10mg/(kg·d),po,qd) | Azithromycin (sequential therapy:10mg/(kg·d),ivgtt,qd and 10mg/(kg·d),po,qd) | 35 | 35 | 19 | 17 | 16 | 18 | 4.61±1.23 | 4.01±1.42 | 2.69±1.09 | 2.97±1.46 | - | 1 2 3 4 5 10 11 13 |
| Zhang 2018a | SHL granules + Azithromycin | Azithromycin | SHL granules (5g,po,tid) + Azithromycin (8mg/(kg·d),po,qd) | Azithromycin (8mg/(kg·d),po,qd) | 65 | 65 | 34 | 35 | 31 | 30 | 6.7±2.2 | 6.8±2.5 | 6.5±1.4 | 6.8±1.5 | 5 | 1 2 3 4 10 |
| Zhang 2018b | SHL granules + Azithromycin | Azithromycin | SHL granules (5g,po,tid) + Azithromycin (8mg/(kg·d),po,qd) | Azithromycin (8mg/(kg·d),po,qd) | 40 | 40 | 45 |  | 35 |  | 4-13 | 4-13 | 3-11 | 3-11 | - | 1 6 7 9 13 |
| Zhao 2015 | SHL granules + Azithromycin | Azithromycin | SHL granules (5g,po,tid) + Azithromycin (10mg/(kg·d),po,qd) | Azithromycin (10mg/(kg·d),po,qd) | 40 | 40 | 56 |  | 24 |  | 7.89±2.1 | 7.89±2.1 | 5-13 | 5-13 | - | 1 |
| Zheng 2015 | SHL granules + Azithromycin | Azithromycin | SHL granules (5g,po,tid) + Azithromycin (10mg/(kg·d),po,qd) | Azithromycin (10mg/(kg·d),po,qd) | 35 | 35 | 18 | 20 | 17 | 15 | 8.63±2.45 | 8.93±2.91 | 8.33±2.16 | 7.67±2.01 | - | 1 2 3 4 6 7 9 13 |

**Groups:** T: Treatment group; C: Control group

**Interventions:** SHL: Shuanghuanlian (oral liquid/ granules)

**Outcome measurements:** 1: response rate; 2: disappearance time of fever; 3: disappearance time of cough; 4: disappearance time of pulmonary rales; 5: average hospitalization time; 6: CD3+ T lymphocytes (CD3+); 7: CD4 + T lymphocytes (CD4+); 8: CD8+ T lymphocytes (CD8+); 9: CD4+ T lymphocytes / CD8+ T lymphocytes (CD4+/CD8+); 10: [interleukin](javascript:;)-6 (IL-6); 11: [interleukin](javascript:;)-8 (IL-8); 12: tumor necrosis factor-α (TNF-α); 13: adverse events.
