# Supplementary material for: Shuanghuanglian oral preparations combined with azithromycin for treatment of Mycoplasma pneumoniae pneumonia in Asian children: A systematic review and meta-analysis of randomized controlled trials
Source: PLoS One. 2021 Jul 13;16(7):e0254405. doi: 10.1371/journal.pone.0254405 (PMC8277054; doi:10.1371/journal.pone.0254405)
Supplement: S2 Table — (DOCX) [file pone.0254405.s009.docx]

# S2 Table. The results of subgroup analysis

| **Outcomes** | **Category** | **P** | **I^2^** |
| --- | --- | --- | --- |
| Response rate | course of the disease | 0.39 | 0% |
| Disappearance time of cough | age | 0.0002 | 93% |
|  | course of disease | 0.0001 | 93.20% |
|  | drug delivery way | 0.02 | 82.30% |
| Disappearance time of pulmonary rales | age | 0.0004 | 92.10% |
|  | course of disease | 0.16 | 49.70% |
|  | drug delivery way | 0.00004 | 92.10% |
