# Supplementary material for: Shuanghuanglian oral preparations combined with azithromycin for treatment of Mycoplasma pneumoniae pneumonia in Asian children: A systematic review and meta-analysis of randomized controlled trials
Source: PLoS One. 2021 Jul 13;16(7):e0254405. doi: 10.1371/journal.pone.0254405 (PMC8277054; doi:10.1371/journal.pone.0254405)
Supplement: S3 Table — Groups: T: Treatment group; C: Control group; Interventions: SHL: Shuanghuanlian (oral liquid/ granules). (DOCX) [file pone.0254405.s010.docx]

**S3 Table. Adverse events**

| ID | Intervention（T/C） |  | adverse events (T) | Sample Size (T) | adverse events (C) | Sample Size (C) | abdominal pain and diarrhea (T/C) | nausea and vomiting (T/C) | Unspecified gastrointestinal reactions (T/C) | Dyspepsia (T/C) | Abnormality of liver and kidney (T/C) | Skin rash (T/C) | Cholestatic jaundice (T/C) | Drowsiness (T/C) | local injection Pain (T/C) | Pericarditis (T/C) | Aseptic meningitis (T/C) |
| --- | --- | --- | --- | --- | --- | --- | --- | --- | --- | --- | --- | --- | --- | --- | --- | --- | --- |
| Fan 2017 | SHL oral liquid + Azithromycin | Azithromycin | 5 | 58 | 7 | 58 |  |  | 2/3 |  |  | 1/2 |  |  | 2/2 |  |  |
| Guo 2017 | SHL oral liquid + Azithromycin | Azithromycin | 7 | 60 | 9 | 60 | 2/3 | 3/3 |  |  | 0/1 | 2/2 |  |  |  |  |  |
| Li 2015 | SHL granules + Azithromycin | Azithromycin | 14 | 100 | 39 | 100 | 3/10 | 5/14 |  |  | 3/9 | 3/6 |  |  |  |  |  |
| Li 2016a | SHL granules + Azithromycin | Azithromycin | 3 | 39 | 11 | 39 | 1/4 | 1/5 |  |  |  | 1/2 |  |  |  |  |  |
| Li 2019 | SHL granules + Azithromycin | Azithromycin | 4 | 40 | 14 | 40 | 2/6 | 1/3 |  |  | 0/3 | 1/2 |  |  |  |  |  |
| Liu 2016 | SHL oral liquid + Azithromycin | Azithromycin | 3 | 50 | 5 | 50 | 1/2 | 2/3 |  |  |  |  |  |  |  |  |  |
| Liu 2018 | SHL oral liquid + Azithromycin | Azithromycin | 6 | 37 | 5 | 37 | 3/1 | 2/2 |  |  |  | 1/2 |  |  |  |  |  |
| Lu 2017 | SHL granules + Azithromycin | Azithromycin | 11 | 78 | 30 | 78 | 5/6 | 2/6 |  | 0/4 |  | 4/7 | 0/2 | 0/5 |  |  |  |
| Wang 2017 | SHL granules + Azithromycin | Azithromycin | 2 | 45 | 6 | 45 | 0/1 | 1/4 |  |  |  | 1/1 |  |  |  |  |  |
| Wang 2018b | SHL oral liquid + Azithromycin | Azithromycin | 1 | 55 | 0 | 55 | 1/0 |  |  |  |  |  |  |  |  |  |  |
| Wang 2018c | SHL oral liquid + Azithromycin | Azithromycin | 3 | 86 | 2 | 86 | 1/1 | 2/1 |  |  |  |  |  |  |  |  |  |
| Wang 2019 | SHL granules + Azithromycin | Azithromycin | 5 | 91 | 3 | 91 | 3/1 | 2/2 |  |  |  |  |  |  |  |  |  |
| Xu 2016 | SHL granules + Azithromycin | Azithromycin | 5 | 40 | 16 | 40 |  |  |  |  |  |  |  |  |  |  |  |
| Yao 2018 | SHL oral liquid + Azithromycin | Azithromycin | 3 | 34 | 4 | 34 | 1/0 | 0/4 |  |  |  | 2/0 |  |  |  |  |  |
| Zhang 2016 | SHL granules + Azithromycin | Azithromycin | 4 | 51 | 13 | 51 | 2/7 | 1/3 |  |  |  | 1/3 |  |  |  |  |  |
| Zhang 2017 | SHL oral liquid + Azithromycin | Azithromycin | 5 |  | 7 |  | 2/3 | 2/2 |  |  |  | 1/2 |  |  |  |  |  |
| Zhang 2018b | SHL granules + Azithromycin | Azithromycin | 5 | 40 | 16 | 40 | 4/8 | 1/2 |  |  |  |  |  |  |  | 0/4 | 0/2 |
| Zheng 2015 | SHL granules + Azithromycin | Azithromycin | 5 | 35 | 14 | 35 | 1/5 | 2/4 |  |  | 1/3 | 1/2 |  |  |  |  |  |

**Groups:** T: Treatment group; C: Control group

**Interventions:** SHL: Shuanghuanlian (oral liquid/ granule)
