# Supplementary material for: Shuanghuanglian oral preparations combined with azithromycin for treatment of Mycoplasma pneumoniae pneumonia in Asian children: A systematic review and meta-analysis of randomized controlled trials
Source: PLoS One. 2021 Jul 13;16(7):e0254405. doi: 10.1371/journal.pone.0254405 (PMC8277054; doi:10.1371/journal.pone.0254405)
Supplement: S1 File — (DOCX) [file pone.0254405.s011.docx]

# S1 File. The detailed search strategy

**The PubMed database Search Strategy**

#1: (((((Mycoplasma pneumoniae pneumonia[MeSH Terms]) OR (Mycoplasma pneumoniae pneumonia[Title/Abstract])) OR (Mycoplasma pneumoniae pneumonia in children[Title/Abstract])) OR (Mycoplasma pneumonia in children[Title/Abstract])) OR (Mycoplasma pneumoniae in pediatric patients[Title/Abstract])) OR (Children with Mycoplasma Pneumonia[Title/Abstract])

Results 4578

#2: ((((Shuanghuanglian[Title/Abstract]) OR (Shuanghuanglian preparations[Title/Abstract])) OR (Shuanghuanglian oral liquid[Title/Abstract])) OR (Shuanguanlian granules [Title/Abstract])) OR (Shuanghuanglian capsule [Title/Abstract])

Results 146

#3: (((((((((randomized controlled study[Title/Abstract]) OR randomized controlled trial[Title/Abstract]) OR randomized trial[Title/Abstract]) OR randomized study[Title/Abstract]) OR randomized placebo-controlled study[Title/Abstract]) OR randomized parallel-group study[Title/Abstract]) OR controlled clinical trial[Title/Abstract]) OR multicenter study[Title/Abstract]) OR double-blinded controlled study[Title/Abstract])

Results 193844

#4：#1 AND #2 AND #3

Results **0**

**The Cochrane library database Search Strategy**

#1: (Mycoplasma pneumoniae pneumonia or Mycoplasma pneumoniae pneumonia in children or Mycoplasma pneumonia in children or Mycoplasma pneumoniae in pediatric patients or Children with Mycoplasma Pneumonia):ti,ab,kw

Results 188

#2: (Shuanghuanglian or SHL or Shuanghuanglian preparations or Shuanghuanglian oral liquid or Shuanguanlian granules or Shuanghuanglian capsule):ti,ab,kw

Results 32

#3: (randomized controlled study or randomized controlled trial or randomized trial or randomized study or randomized placebo-controlled study or randomized parallel-group study or controlled clinical trial or multicenter study or double-blinded controlled study):ti,ab,kw

Results 894596

#:4：#1 AND #2 AND#3

Results 0

**The Embase database Search Strategy**

#1: 'mycoplasma pneumoniae pneumonia':ab,ti OR 'mycoplasma pneumoniae pneumonia in children':ab,ti OR 'mycoplasma pneumonia in children':ab,ti OR 'mycoplasma pneumoniae in pediatric patients':ab,ti OR 'children with mycoplasma pneumonia':ab,ti

Results 665

#2: ' Shuanghuanglian ':ab,ti OR 'shuanghuanglian oral liquid':ab,ti OR ' Shuanghuanglian preparations ':ab,ti OR ' Shuanguanlian granules ':ab,ti OR ' Shuanguanlian capsule ':ab,ti

Results 1356

#3: 'randomized controlled study':ab,ti OR 'randomized controlled trial':ab,ti OR 'randomized trial':ab,ti OR 'randomized study':ab,ti OR 'randomized placebo-controlled study':ab,ti OR 'randomized parallel-group study':ab,ti OR 'controlled clinical trial':ab,ti OR 'multicenter study':ab,ti OR 'double-blinded controlled study':ab,ti

Results 172635

#4: #1 AND #2 AND #3

Results 0

**MEDILINE database Search Strategy**

#1
SU mycoplasma pneumoniae pneumonia OR SU mycoplasma pneumoniae pneumonia in children OR SU mycoplasma pneumonia in children OR SU mycoplasma pneumoniae in pediatric patients OR SU children with mycoplasma pneumonia

Results 4630

#2
SU Shuanghuanglian OR TX shuanghuanglian oral liquid OR TX Shuanghuanglian preparations OR TX Shuanguanlian granules OR TX Shuanguanlian capsule

Results 43

#3
AB randomized controlled study OR AB randomized controlled trial OR AB randomized trial OR AB randomized study OR AB randomized placebo-controlled study OR AB randomized parallel-group study OR AB controlled clinical trial OR AB multicenter study OR AB double-blinded controlled study

Results 914408

#4: #1 AND #2 AND #3

Results 0

**Results of the references of included studies and reviews and Register the website of clinical trials**

References of included studied Results =0

References of reviews Results =0

Chinese Clinical Trial Registry (http://www.chictr.org.cn) Results =0
