# Supplementary material for: Shuanghuanglian oral preparations combined with azithromycin for treatment of Mycoplasma pneumoniae pneumonia in Asian children: A systematic review and meta-analysis of randomized controlled trials
Source: PLoS One. 2021 Jul 13;16(7):e0254405. doi: 10.1371/journal.pone.0254405 (PMC8277054; doi:10.1371/journal.pone.0254405)
Supplement: S1 Dataset — (DOCX) [file pone.0254405.s012.docx]

**S1 Dataset. Datasets of all outcomes and safety**

**Response Rate**

| study | t | c | rt | nt | rc | nc |
| --- | --- | --- | --- | --- | --- | --- |
| Fan 2017 | SHL+Azithromycin | Azithromycin | 55 | 58 | 47 | 58 |
| Guo 2017 | SHL+Azithromycin | Azithromycin | 56 | 60 | 45 | 60 |
| Li 2015 | SHL+Azithromycin | Azithromycin | 91 | 100 | 84 | 100 |
| Li 2016a | SHL+Azithromycin | Azithromycin | 37 | 39 | 29 | 39 |
| Li 2016b | SHL+Azithromycin | Azithromycin | 91 | 100 | 84 | 100 |
| Li 2016c | SHL+Azithromycin | Azithromycin | 34 | 35 | 29 | 35 |
| Li 2019 | SHL+Azithromycin | Azithromycin | 38 | 40 | 31 | 40 |
| Liu 2016 | SHL+Azithromycin | Azithromycin | 50 | 50 | 48 | 50 |
| Liu 2018 | SHL+Azithromycin | Azithromycin | 37 | 37 | 36 | 37 |
| Liu 2019 | SHL+Azithromycin | Azithromycin | 57 | 60 | 49 | 60 |
| Lu 2017 | SHL+Azithromycin | Azithromycin | 74 | 78 | 60 | 78 |
| Lu 2018 | SHL+Azithromycin | Azithromycin | 48 | 49 | 46 | 49 |
| Luo 2017 | SHL+Azithromycin | Azithromycin | 58 | 60 | 50 | 60 |
| Shi 2020 | SHL+Azithromycin | Azithromycin | 38 | 39 | 32 | 39 |
| Wang 2017 | SHL+Azithromycin | Azithromycin | 44 | 45 | 38 | 45 |
| Wang 2018a | SHL+Azithromycin | Azithromycin | 20 | 20 | 15 | 20 |
| Wang 2018b | SHL+Azithromycin | Azithromycin | 51 | 55 | 43 | 55 |
| Wang 2018c | SHL+Azithromycin | Azithromycin | 83 | 86 | 76 | 86 |
| Wang 2019 | SHL+Azithromycin | Azithromycin | 89 | 91 | 80 | 91 |
| Xu 2016 | SHL+Azithromycin | Azithromycin | 37 | 40 | 31 | 40 |
| Yao 2018 | SHL+Azithromycin | Azithromycin | 33 | 34 | 25 | 34 |
| Zhang 2016 | SHL+Azithromycin | Azithromycin | 50 | 51 | 41 | 51 |
| Zhang 2017 | SHL+Azithromycin | Azithromycin | 34 | 35 | 27 | 35 |
| Zhang 2018a | SHL+Azithromycin | Azithromycin | 62 | 65 | 53 | 65 |
| Zhang 2018b | SHL+Azithromycin | Azithromycin | 37 | 40 | 31 | 40 |
| Zhao 2015 | SHL+Azithromycin | Azithromycin | 38 | 40 | 29 | 40 |
| Zheng 2015 | SHL+Azithromycin | Azithromycin | 32 | 35 | 27 | 35 |

**Disappearance time of cough**

| study | t | c | mdt | sdt | nt | mdc | sdc | nc |
| --- | --- | --- | --- | --- | --- | --- | --- | --- |
| Li 2015 | SHL+Azithromycin | Azithromycin | 4.9 | 1.7 | 100 | 8.4 | 0.3 | 100 |
| Li 2016a | SHL+Azithromycin | Azithromycin | 4.57 | 1.72 | 39 | 8.14 | 1.85 | 39 |
| Li 2016c | SHL+Azithromycin | Azithromycin | 4.5 | 1.1 | 35 | 7.4 | 1.6 | 35 |
| Li 2019 | SHL+Azithromycin | Azithromycin | 3.83 | 1.25 | 40 | 6.12 | 1.43 | 40 |
| Liu 2018 | SHL+Azithromycin | Azithromycin | 4.37 | 1.24 | 37 | 6.13 | 2.21 | 37 |
| Liu 2019 | SHL+Azithromycin | Azithromycin | 5.22 | 1.21 | 60 | 8.37 | 2.08 | 60 |
| Lu 2017 | SHL+Azithromycin | Azithromycin | 3.75 | 0.52 | 78 | 8.24 | 0.61 | 78 |
| Shi 2020 | SHL+Azithromycin | Azithromycin | 4.33 | 1.6 | 39 | 6 | 2.03 | 39 |
| Wang 2018b | SHL+Azithromycin | Azithromycin | 7.04 | 2.14 | 55 | 9.63 | 2.74 | 55 |
| Xu 2016 | SHL+Azithromycin | Azithromycin | 4.48 | 2.26 | 40 | 8.56 | 2.53 | 40 |
| Yao 2018 | SHL+Azithromycin | Azithromycin | 4.87 | 1.31 | 34 | 6.99 | 1.43 | 34 |
| Zhang 2017 | SHL+Azithromycin | Azithromycin | 4.53 | 1.59 | 35 | 5.91 | 2.07 | 35 |
| Zhang 2018a | SHL+Azithromycin | Azithromycin | 4.75 | 2.06 | 65 | 8.56 | 2.52 | 65 |
| Zheng 2015 | SHL+Azithromycin | Azithromycin | 4.59 | 2.15 | 35 | 8.45 | 2.64 | 35 |

**Disappearance time of fever**

| study | t | c | mdt | sdt | nt | mdc | sdc | nc |
| --- | --- | --- | --- | --- | --- | --- | --- | --- |
| Li 2015 | SHL+Azithromycin | Azithromycin | 2.2 | 0.8 | 100 | 3.8 | 0.2 | 100 |
| Li 2016a | SHL+Azithromycin | Azithromycin | 3.93 | 1.54 | 39 | 5.87 | 1.61 | 39 |
| Li 2016c | SHL+Azithromycin | Azithromycin | 2.8 | 0.6 | 35 | 4.2 | 0.8 | 35 |
| Li 2019 | SHL+Azithromycin | Azithromycin | 3.43 | 1.86 | 40 | 5.37 | 2.25 | 40 |
| Liu 2018 | SHL+Azithromycin | Azithromycin | 2.79 | 1.12 | 37 | 4.13 | 1.32 | 37 |
| Liu 2019 | SHL+Azithromycin | Azithromycin | 2.15 | 0.48 | 60 | 4.24 | 1.19 | 60 |
| Lu 2017 | SHL+Azithromycin | Azithromycin | 2.51 | 0.38 | 78 | 6.72 | 0.54 | 78 |
| Shi 2020 | SHL+Azithromycin | Azithromycin | 2.08 | 1.01 | 39 | 3 | 1.1 | 39 |
| Wang 2018a | SHL+Azithromycin | Azithromycin | 1.5 | 1.1 | 20 | 3.1 | 1 | 20 |
| Wang 2018b | SHL+Azithromycin | Azithromycin | 2.93 | 1.08 | 55 | 3.84 | 1.33 | 55 |
| Xu 2016 | SHL+Azithromycin | Azithromycin | 3.71 | 1.52 | 40 | 5.68 | 1.7 | 40 |
| Yao 2018 | SHL+Azithromycin | Azithromycin | 2.41 | 1.98 | 34 | 3.83 | 1.76 | 34 |
| Zhang 2017 | SHL+Azithromycin | Azithromycin | 2.11 | 1.03 | 35 | 2.98 | 1.13 | 35 |
| Zhang 2018a | SHL+Azithromycin | Azithromycin | 3.88 | 1.5 | 65 | 5.65 | 1.72 | 65 |
| Zheng 2015 | SHL+Azithromycin | Azithromycin | 3.82 | 1.63 | 35 | 5.57 | 1.81 | 35 |

**Disappearance time of pulmonary rale**

| study | t | c | mdt | sdt | nt | mdc | sdc | nc |
| --- | --- | --- | --- | --- | --- | --- | --- | --- |
| Li 2016c | SHL+Azithromycin | Azithromycin | 6.3 | 1.2 | 35 | 8.5 | 1.9 | 35 |
| Liu 2018 | SHL+Azithromycin | Azithromycin | 5.82 | 1.21 | 37 | 6.95 | 2.11 | 37 |
| Liu 2019 | SHL+Azithromycin | Azithromycin | 4.03 | 0.96 | 60 | 7.21 | 1.93 | 60 |
| Lu 2017 | SHL+Azithromycin | Azithromycin | 5.13 | 0.47 | 78 | 8.82 | 0.51 | 78 |
| Shi 2020 | SHL+Azithromycin | Azithromycin | 3.68 | 1.33 | 39 | 4.91 | 1.92 | 39 |
| Wang 2018b | SHL+Azithromycin | Azithromycin | 8.34 | 1.83 | 55 | 10.76 | 2.05 | 55 |
| Yao 2018 | SHL+Azithromycin | Azithromycin | 3.97 | 1.12 | 34 | 5.09 | 2.01 | 34 |
| Zhang 2017 | SHL+Azithromycin | Azithromycin | 3.71 | 1.35 | 35 | 4.89 | 1.93 | 35 |
| Zhang 2018a | SHL+Azithromycin | Azithromycin | 4.25 | 1.52 | 65 | 6.8 | 1.88 | 65 |
| Zheng 2015 | SHL+Azithromycin | Azithromycin | 4.37 | 1.62 | 35 | 6.71 | 1.79 | 35 |

**Average hospitalization time**

| study | t | c | mdt | sdt | nt | mdc | sdc | nc |
| --- | --- | --- | --- | --- | --- | --- | --- | --- |
| Li 2015 | SHL+Azithromycin | Azithromycin | 12.5 | 0.8 | 100 | 15.7 | 1.6 | 100 |
| Li 2019 | SHL+Azithromycin | Azithromycin | 8.71 | 2.38 | 40 | 12.73 | 2.86 | 40 |
| Liu 2019 | SHL+Azithromycin | Azithromycin | 6.26 | 1.33 | 60 | 11.93 | 3.09 | 60 |
| Lu 2017 | SHL+Azithromycin | Azithromycin | 10.42 | 1.78 | 78 | 14.31 | 1.07 | 78 |
| Wang 2018a | SHL+Azithromycin | Azithromycin | 7 | 2 | 20 | 12.8 | 3.3 | 20 |
| Xu 2016 | SHL+Azithromycin | Azithromycin | 14.12 | 2.88 | 40 | 19.56 | 3.44 | 40 |
| Zhang 2017 | SHL+Azithromycin | Azithromycin | 8.49 | 2.38 | 35 | 10.58 | 1.96 | 35 |

**CD3+ T-lymphocytes**

| study | t | c | mdt | sdt | nt | mdc | sdc | nc |
| --- | --- | --- | --- | --- | --- | --- | --- | --- |
| Guo 2017 | SHL+Azithromycin | Azithromycin | 15.3 | 11.98791058 | 60 | 8.2 | 10.83928042 | 60 |
| Liu 2016 | SHL+Azithromycin | Azithromycin | 9.7 | 2.6 | 50 | 4 | 1.7 | 50 |
| Lu 2018 | SHL+Azithromycin | Azithromycin | 9.89 | 5.234873446 | 49 | 5.08 | 4.668072407 | 49 |
| Wang 2017 | SHL+Azithromycin | Azithromycin | 18.1 | 8.64175908 | 45 | 6.5 | 7.238093672 | 45 |
| Wang 2018c | SHL+Azithromycin | Azithromycin | 13.25 | 3.98086674 | 86 | 9.14 | 3.930686963 | 86 |
| Wang 2019 | SHL+Azithromycin | Azithromycin | 9.88 | 5.251866335 | 91 | 5.05 | 4.691172561 | 91 |
| Zhang 2016 | SHL+Azithromycin | Azithromycin | 16.9 | 8.229216245 | 51 | 6.8 | 7.917070165 | 51 |
| Zhang 2018b | SHL+Azithromycin | Azithromycin | 17.9 | 9.482088378 | 40 | 9.3 | 7.66093989 | 40 |
| Zheng 2015 | SHL+Azithromycin | Azithromycin | 15.9 | 8.471717653 | 35 | 7.3 | 7.662245102 | 35 |

**CD4+ T-lymphocytes**

| study | t | c | mdt | sdt | nt | mdc | sdc | nc |
| --- | --- | --- | --- | --- | --- | --- | --- | --- |
| Guo 2017 | SHL+Azithromycin | Azithromycin | 6.8 | 0.778716893 | 60 | 4 | 0.661588996 | 60 |
| Li 2019 | SHL+Azithromycin | Azithromycin | 8.71 | 2.708375897 | 40 | 3.24 | 2.797909934 | 40 |
| Liu 2016 | SHL+Azithromycin | Azithromycin | 8.2 | 0.9 | 50 | 3.1 | 0.3 | 50 |
| Lu 2018 | SHL+Azithromycin | Azithromycin | 8.73 | 2.197248279 | 49 | 2.34 | 2.076318858 | 49 |
| Wang 2017 | SHL+Azithromycin | Azithromycin | 9 | 4.942671343 | 45 | 3.2 | 3.659234893 | 45 |
| Wang 2018c | SHL+Azithromycin | Azithromycin | 12.19 | 1.728698933 | 86 | 7.62 | 1.850864663 | 86 |
| Wang 2019 | SHL+Azithromycin | Azithromycin | 8.72 | 2.230313879 | 91 | 2.36 | 2.096210867 | 91 |
| Zhang 2016 | SHL+Azithromycin | Azithromycin | 8.8 | 5.901694672 | 51 | 5.1 | 5.5 | 51 |
| Zhang 2018b | SHL+Azithromycin | Azithromycin | 8.2 | 6.255397669 | 40 | 4.9 | 4.550824101 | 40 |
| Zheng 2015 | SHL+Azithromycin | Azithromycin | 8.2 | 5.703507693 | 35 | 5 | 5.650663678 | 35 |

**CD8+ T-lymphocytes**

| study | t | c | mdt | sdt | nt | mdc | sdc | nc |
| --- | --- | --- | --- | --- | --- | --- | --- | --- |
| Li 2019 | SHL+Azithromycin | Azithromycin | -6.06 | 1.915646105 | 40 | -3.08 | 2.061140461 | 40 |
| Liu 2016 | SHL+Azithromycin | Azithromycin | -8.1 | 1.1 | 50 | -3.3 | 1.3 | 50 |
| Lu 2018 | SHL+Azithromycin | Azithromycin | -8.86 | 1.947998973 | 49 | -3.49 | 1.972891279 | 49 |
| Wang 2018c | SHL+Azithromycin | Azithromycin | -7.84 | 1.645843249 | 86 | -3.88 | 1.650817979 | 86 |
| Wang 2019 | SHL+Azithromycin | Azithromycin | -8.86 | 1.966494343 | 91 | -3.48 | 2.004719432 | 91 |

**CD4+/CD8+**

| study | t | c | mdt | sdt | nt | mdc | sdc | nc |
| --- | --- | --- | --- | --- | --- | --- | --- | --- |
| Guo 2017 | SHL+Azithromycin | Azithromycin | 0.4 | 0.339558537 | 60 | 0.31 | 0.401497198 | 60 |
| Li 2019 | SHL+Azithromycin | Azithromycin | 0.53 | 0.359304884 | 40 | 0.29 | 0.315753068 | 40 |
| Wang 2017 | SHL+Azithromycin | Azithromycin | 0.6 | 0.360555128 | 45 | 0.1 | 0.264575131 | 45 |
| Wang 2018c | SHL+Azithromycin | Azithromycin | 0.83 | 0.373630834 | 86 | 0.24 | 0.314324673 | 86 |
| Zhang 2016 | SHL+Azithromycin | Azithromycin | 0.56 | 0.453982379 | 51 | 0.34 | 0.545802162 | 51 |
| Zhang 2018b | SHL+Azithromycin | Azithromycin | 0.54 | 0.550726793 | 40 | 0.31 | 0.476550102 | 40 |
| Zheng 2015 | SHL+Azithromycin | Azithromycin | 0.54 | 0.4592385 | 35 | 0.31 | 0.572974694 | 35 |

**Interleukin-6**

| study | t | c | mdt | sdt | nt | mdc | sdc | nc |
| --- | --- | --- | --- | --- | --- | --- | --- | --- |
| Fan 2017 | SHL+Azithromycin | Azithromycin | -8.8 | 3.031501278 | 58 | -5.7 | 3.439476704 | 58 |
| Li 2016b | SHL+Azithromycin | Azithromycin | -41.5 | 4.01497198 | 100 | -23.7 | 3.651027253 | 100 |
| Li 2019 | SHL+Azithromycin | Azithromycin | -8.9 | 3.992380242 | 40 | -4.02 | 3.746051254 | 40 |
| Liu 2016 | SHL+Azithromycin | Azithromycin | -8.2 | 3.3 | 50 | -3.2 | 1.7 | 50 |
| Liu 2018 | SHL+Azithromycin | Azithromycin | -8.7 | 3.304542328 | 37 | -2.4 | 5.121523211 | 37 |
| Lu 2018 | SHL+Azithromycin | Azithromycin | -8.12 | 4.377978986 | 49 | -3.22 | 5.671357862 | 49 |
| Wang 2018c | SHL+Azithromycin | Azithromycin | -41.94 | 10.28641337 | 86 | -28.69 | 11.04571863 | 86 |
| Wang 2019 | SHL+Azithromycin | Azithromycin | -8.11 | 4.381746227 | 91 | -3.23 | 5.67891715 | 91 |
| Zhang 2017 | SHL+Azithromycin | Azithromycin | -10.34 | 2.065236064 | 35 | -5.08 | 2.078051972 | 35 |
| Zhang 2018a | SHL+Azithromycin | Azithromycin | -43.57 | 3.888791586 | 65 | -25.29 | 3.3186895 | 65 |

**Interleukin-8**

| study | t | c | mdt | sdt | nt | mdc | sdc | nc |
| --- | --- | --- | --- | --- | --- | --- | --- | --- |
| Fan 2017 | SHL+Azithromycin | Azithromycin | -9.9 | 3.204684072 | 58 | -5.1 | 3.593048845 | 58 |
| Li 2019 | SHL+Azithromycin | Azithromycin | -31.95 | 5.069625233 | 40 | -29.92 | 5.458268956 | 40 |
| Liu 2016 | SHL+Azithromycin | Azithromycin | -36.7 | 3.3 | 50 | -32.1 | 3.8 | 50 |
| Liu 2018 | SHL+Azithromycin | Azithromycin | -35.9 | 4.439594576 | 37 | -30.2 | 4.085339643 | 37 |
| Lu 2018 | SHL+Azithromycin | Azithromycin | -34.93 | 4.564438629 | 49 | -31.49 | 4.167457258 | 49 |
| Zhang 2017 | SHL+Azithromycin | Azithromycin | -11.58 | 2.942668857 | 35 | -6.67 | 2.646148144 | 35 |

**TNF-α**

| study | t | c | mdt | sdt | nt | mdc | sdc | nc |
| --- | --- | --- | --- | --- | --- | --- | --- | --- |
| Fan 2017 | SHL+Azithromycin | Azithromycin | -8.9 | 4.744470466 | 58 | -3.7 | 5.534437641 | 58 |
| Li 2019 | SHL+Azithromycin | Azithromycin | -31.47 | 5.997299392 | 40 | -20.56 | 5.933363296 | 40 |
| Liu 2016 | SHL+Azithromycin | Azithromycin | -30.2 | 3.4 | 50 | -21.1 | 4.2 | 50 |
| Liu 2018 | SHL+Azithromycin | Azithromycin | -28.4 | 5.1 | 37 | -20.5 | 4.246174749 | 37 |
| Lu 2018 | SHL+Azithromycin | Azithromycin | -29.5 | 4.345077675 | 49 | -20.9 | 4.822209037 | 49 |
| Wang 2018c | SHL+Azithromycin | Azithromycin | -69.88 | 19.89215675 | 86 | -47.64 | 22.46056767 | 86 |
| Wang 2019 | SHL+Azithromycin | Azithromycin | -29.5 | 4.362235665 | 91 | -21.2 | 4.83550411 | 91 |

**Adverse rate**

| study | t | c | rt | nt | rc | nc |
| --- | --- | --- | --- | --- | --- | --- |
| Fan 2017 | SHL+Azithromycin | Azithromycin | 5 | 58 | 7 | 58 |
| Guo 2017 | SHL+Azithromycin | Azithromycin | 7 | 60 | 9 | 60 |
| Li 2015 | SHL+Azithromycin | Azithromycin | 14 | 100 | 39 | 100 |
| Li 2016a | SHL+Azithromycin | Azithromycin | 3 | 39 | 11 | 39 |
| Li 2019 | SHL+Azithromycin | Azithromycin | 4 | 40 | 14 | 40 |
| Liu 2016 | SHL+Azithromycin | Azithromycin | 3 | 50 | 5 | 50 |
| Liu 2018 | SHL+Azithromycin | Azithromycin | 6 | 37 | 5 | 37 |
| Lu 2017 | SHL+Azithromycin | Azithromycin | 11 | 78 | 30 | 78 |
| Wang 2017 | SHL+Azithromycin | Azithromycin | 2 | 45 | 6 | 45 |
| Wang 2018b | SHL+Azithromycin | Azithromycin | 1 | 55 | 0 | 55 |
| Wang 2018c | SHL+Azithromycin | Azithromycin | 3 | 86 | 2 | 86 |
| Wang 2019 | SHL+Azithromycin | Azithromycin | 5 | 91 | 3 | 91 |
| Xu 2016 | SHL+Azithromycin | Azithromycin | 5 | 40 | 16 | 40 |
| Yao 2018 | SHL+Azithromycin | Azithromycin | 3 | 34 | 4 | 34 |
| Zhang 2016 | SHL+Azithromycin | Azithromycin | 4 | 51 | 13 | 51 |
| Zhang 2018b | SHL+Azithromycin | Azithromycin | 5 | 40 | 16 | 40 |
| Zheng 2015 | SHL+Azithromycin | Azithromycin | 5 | 35 | 14 | 35 |
